# IFN-I-mediated neutropoiesis bias drives neutrophil priming and inflammatory comorbidities

**DOI:** 10.7150/thno.110859

**Published:** 2025-05-06

**Authors:** Yuman Li, Yiming Chen, Chenyu Deng, Yuting Niu, Yue Yang, Shiyu Sun, Zhewen Hu, Yan Wei, Mingming Xu, Ying Huang, Thomas Van Dyke, Xuliang Deng

**Affiliations:** 1 Department of Geriatric Dentistry, Peking University School and Hospital of Stomatology & National Center for Stomatology & National Clinical Research Center for Oral Diseases & National Engineering Research Center of Oral Biomaterials and Digital Medical Devices, Beijing, 100081, PR China.; 2 Department of Prosthodontics, The First Clinical Division, Peking University School and Hospital of Stomatology, Beijing, 100034, PR China.; 3 Department of Inflammation and Immunology, ADA Forsyth Institute, Somerville, MA 02143, USA.

**Keywords:** periodontitis, inflammatory comorbidities, neutropoiesis, hematopoietic stem and progenitor cells, single-cell multiomics sequencing

## Abstract

**Rationale:** Local chronic inflammation is increasingly recognized as a driver of systemic inflammatory comorbidities; however, the underlying mechanisms remain incompletely understood. This study investigates the impact of periodontitis on the reprogramming of bone marrow hematopoiesis, with a focus on neutropoiesis bias and its contribution to the exacerbation of arthritis.

**Methods:** Single-cell multiomics sequencing was performed on hematopoietic stem and progenitor cells (HSPCs) isolated from control and ligature-induced periodontitis (LIP) mice to characterize transcriptional and epigenetic alterations. Differentiation trajectories and key transcription factors (TFs) governing neutrophil lineage commitment were identified. Neutrophil priming was assessed using Smart-seq2, bulk RNA-seq, and lipopolysaccharide stimulation assays. The functional role of primed neutrophils in arthritis was evaluated through adoptive transfer, *in vivo* tracking, and functional blockade within a collagen antibody-induced arthritis model. Type I interferon (IFN-I) signaling was interrogated using Ifnar1⁻/⁻ mice and neutralizing antibodies to elucidate mechanistic pathways. Reversibility of neutropoiesis bias and arthritis aggravation was examined following ligature removal to model periodontitis resolution.

**Results:** Transcriptional and chromatin accessibility profiling demonstrated that LIP induces a selective skewing of HSPC differentiation toward the neutrophil lineage. This reprogramming results in sustained expansion of primed neutrophils, which contribute to the aggravation of distal arthritis. Mechanistically, elevated IFN-I levels promote continuous neutropoiesis bias through activation of IFN-I signaling in HSPCs. *Rarg* and *Nr2f6* were identified as potential TFs contributing to IFN-I-mediated neutrophil lineage commitment. Notably, resolution of periodontitis reversed the hematopoietic bias and mitigated arthritis progression.

**Conclusions:** Periodontitis exacerbates arthritis through IFN-I-mediated neutropoiesis bias, emphasizing the necessity of controlling local chronic inflammation in the management of systemic inflammatory comorbidities.

## Introduction

Local chronic inflammation is a well-established risk factor for the onset and progression of distal organ comorbidities 1-5. Prominent examples include the link between periodontitis and conditions such as arthritis, cardiovascular disease, type 2 diabetes, and cancer 6-11. As these comorbidities constitute a significant proportion of non-communicable diseases globally, they represent a major public health concern 12-14.

Recent evidence has revealed that innate immune cells can exhibit a form of memory or priming that persists beyond the duration of a localized inflammatory lesion, mediated in part by direct modulation of bone marrow (BM) activity 15. Hematopoietic stem and progenitor cells (HSPCs) act as central coordinators within the BM, responding to peripheral inflammatory cues by expanding and priming myeloid progeny. This process facilitates a feedback mechanism from the BM to peripheral tissues, leading to heightened inflammatory responses driven by activated neutrophils and macrophages 16. Previous models focused on the diffusion of inflammatory mediators from localized sites to systemic circulation as the primary mode of propagation 10, 17. However, emerging insights suggest that HSPCs directly sense inflammatory signals via cytokine receptors and adapt by augmenting proliferation and preferential differentiation toward the myeloid lineage 16, 18, 19. These inflammatory adaptations are crucial for meeting the increased demand for innate immune cells and enhancing responsiveness to subsequent stimuli 20, 21. Nevertheless, when inflammation becomes chronic—as observed in periodontitis—sustained alterations in hematopoiesis result in persistent elevation and activation of innate immune cells, with systemic consequences. Such skewed hematopoietic output may contribute to the development and progression of distal organ comorbidities in the context of persistent local chronic inflammation.

Neutropoiesis is a part of the hematopoietic hierarchical architecture in which HSPCs give rise to highly proliferative, lineage-committed neutrophil progenitors that subsequently differentiate into mature neutrophils 22. In inflammatory disease settings, circulating neutrophils often exhibit an enhanced and accelerated inflammatory response 23-25. These observations support the hypothesis that a specific neutropoiesis bias during hematopoiesis may underlie the mechanism by which local chronic inflammation exacerbates the progression of distal organ comorbidities.

In this study, single-cell multiomics analysis—integrating both scRNA-seq and scATAC-seq from the same cells—was employed to examine the impact of periodontitis on the transcriptomic and epigenomic landscape of HSPCs. The results demonstrate that periodontitis induces a shift in hematopoietic commitment toward the neutrophil lineage, leading to the systemic release of primed neutrophils that exacerbate arthritis. Mechanistically, type I interferons (IFN-I) were identified as key drivers of this neutrophil lineage skewing. Intriguingly, resolution of periodontitis effectively reversed the neutropoiesis bias, normalized circulating neutrophil levels, and alleviated arthritis progression. These findings offer new perspectives on the periodontitis-BM-arthritis axis and highlight how local chronic inflammation can exacerbate distant organ comorbidities, emphasizing the necessity of controlling local chronic inflammation in the management of systemic inflammatory diseases.

## Results

### Periodontitis triggers sustained hematopoietic inflammatory adaptation

**Figure 1:**
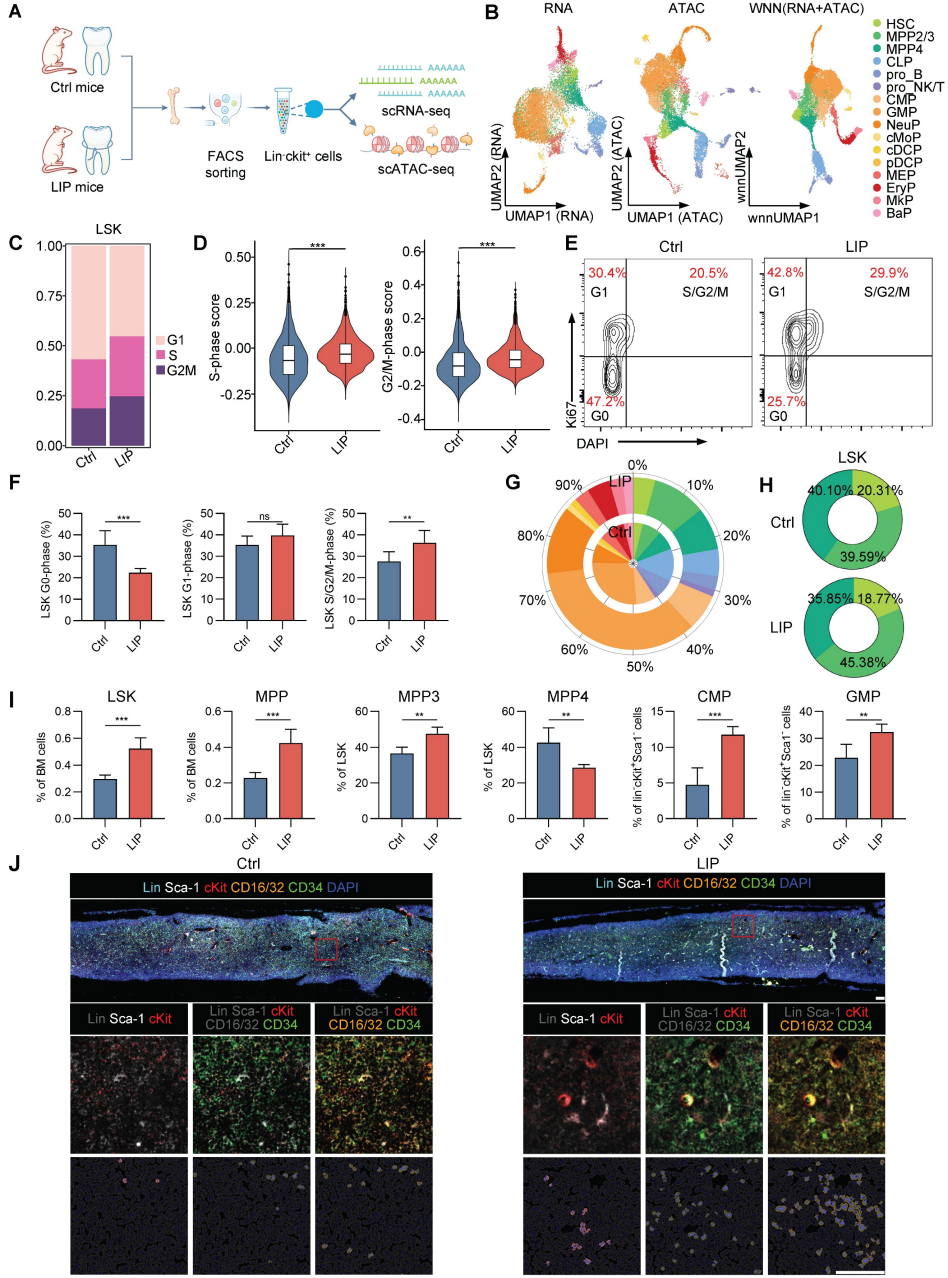
** Periodontitis mediates hematopoietic inflammatory adaptation. (A)** Schematic for single-cell multiomics sequencing of HSPCs (lin^-^cKit^+^) from Ctrl and LIP mice. BM cells from five mice were pooled into a single sample for each group, and lin^-^cKit^+^ cells were sorted via flow cytometry prior to single-cell multiomics sequencing. **(B)** UMAP visualizations were computed using RNA, ATAC, or WNN analysis, respectively. Cell annotations were derived from WNN analysis according to representative markers. **(C)** Repartition (in percentage) of the cell cycle phases (estimated with cyclone) in LSK cells of Ctrl and LIP mice. **(D)** Comparisons of S-phase and G2/M-phase score of LSK cells between Ctrl and LIP mice. **(E-F)** Cell cycle analysis of LSK cells from Ctrl and LIP mice, performed using Ki67 and DAPI staining. (E) Representative flow cytometry plots, and (F) bar graph showing the frequency of LSK cells in different phases of the cell cycle. **(G)** Stacked radial chart depicting the distribution of HSPC subsets from Ctrl (inner circle) and LIP (outer circle) mice, based on single-cell multiomics sequencing data. The colors correspond to the identified cell types. **(H)** Donut plots illustrating the proportion of HSC, MPP2/3, and MPP4 within the LSK cells, also derived from single-cell multiomics sequencing data. The colors represent the respective cell types. **(I)** Frequencies of LSK and MPP in total BM cells. Frequencies of MPP subsets (MPP3 and MPP4) in LSK cells. Frequencies of CMP and GMP in Lin^-^cKit^+^Sca1^-^ cells. **(J)** Representative IF staining showing LSK, CMP, and GMP in the BM of Ctrl (left) and LIP (right) mice (scale bar = 100 µm). Data are presented as the mean ± SD from at least three independent experiments; n = 6 mice/group (F and I). *P* values were calculated using two-tailed Student's *t* test; *P < 0.05, **P < 0.01, ***P < 0.001, ns indicates no significant difference.

To investigate whether experimental periodontitis triggers hematopoietic inflammatory adaptation in the BM, mice were subjected to ligation-induced periodontitis (LIP) for 14 d, while control mice (Ctrl) remained unligated throughout the same period. Single-cell multiomics sequencing—combining scRNA-seq and scATAC-seq from the same cell—was employed to directly correlate transcriptional activity with regulatory accessibility, facilitating a more accurate reconstruction of molecular processes governing cell physiology 26-28. HSPCs (Lin^-^cKit^+^) were isolated from Ctrl and LIP mice for analysis (Figure 1A). Following quality control, 13,527 high-quality single cell transcriptomes and epigenomes were retained, comprising 6,093 cells from the Ctrl group and 7,434 cells from the LIP group. After batch correction, a weighted nearest-neighbor analysis was performed to integrate the RNA expression and chromatin accessibility data, resulting in the identification of 16 distinct cell clusters (Figure 1B and Figure S1A). Projection of these clusters onto the scRNA-seq and scATAC-seq data demonstrated strong concordance (Figure 1B). Within the LSK (Lin^-^cKit^+^Sca1^+^) compartment, subsets corresponding to hematopoietic stem cells (HSC), multipotent progenitor cells 2/3 (MPP2/3) and MPP4 were identified. The progenitor compartment included annotated clusters representing common lymphoid progenitors (CLP), progenitor B cells (pro_B), progenitor NK/T cells (pro_NK/T), common myeloid progenitors (CMP), granulocyte-monocyte progenitors (GMP), neutrophil progenitors (NeuP), monocyte progenitors (cMoP), conventional dendritic cell progenitors (cDCP), plasmacytoid dendritic cell progenitors (pDCP), megakaryocyte-erythrocyte progenitors (MEP), erythrocyte progenitors (EryP), megakaryocyte progenitors (MkP), and basophil progenitors (BaP), as shown in the uniform manifold approximation and projection (UMAP) space (Figure 1B and Figure S1B). Each cell cluster was defined based on the expression and chromatin activity of canonical marker genes (Figure S1C). Cell cycle analysis of LSK cells revealed a significant increase in the proportion of cells in S-phase and G2/M-phase in the LIP group compared with the Ctrl group (Figure 1C). Moreover, genes related to the S-phase and G2/M-phase progression were notably upregulated in LSK cells from the LIP group, indicating enhanced cell cycle activity in HSPCs in response to periodontitis (Figure 1D). Flow cytometry analyses further demonstrated an increased proportion of cells in S/G2/M-phase in the LIP group (Figure 1E-F). These findings collectively suggest that periodontitis stimulates HSPC proliferation as part of a sustained hematopoietic inflammatory adaptation.

Moreover, expansion of the LSK—including HSC, MPP2/3, and MPP4—as well as myeloid progenitors (CMP, GMP, and NeuP), was observed in the LIP group compared to the Ctrl group, whereas lymphoid progenitors (CLP and pro_B) were predominantly derived from Ctrl mice (Figure 1G). Notably, the proportion of the myeloid-biased MPP2/3 subset within the LSK population increased in LIP mice, while the proportions of HSCs and the lymphoid-biased MPP4 subset decreased relative to controls (Figure 1H). These heterogeneous changes in HSPCs were validated by flow cytometry (Figure S1D). LIP-treated mice exhibited significantly increased frequencies and absolute numbers of LSK and MPP (CD48^+^CD150^-^LSK) cells in the BM compared to Ctrl mice on day 14 (Figure 1I and Figure S1E). In contrast, no significant differences were observed in the frequencies or numbers of LT-HSCs (Flt3^-^CD48^-^CD150^+^LSK) or ST-HSCs (Flt3^-^CD48^-^CD150^-^LSK) between groups (Figure S1F). Further analysis of MPP subsets revealed a notable increase in the frequency and number of myeloid-biased MPP3 cells (Flt3^-^CD48^+^CD150^-^LSK), along with a corresponding decrease in lymphoid-biased MPP4 cells (Flt3^+^CD48^+^CD150^-^LSK) in the LIP group. Moreover, LIP mice showed marked expansion of both CMPs (Lin^-^cKit^+^Sca1^-^CD16/32^-^CD34^+^) and GMPs (Lin^-^cKit^+^Sca1^-^CD16/32^+^CD34^+^), accompanied by a significant reduction in CLPs (Lin^-^CD127^+^Sca-1^lo^cKit^lo^) (Figure 1I and Figure S1E-F). Multicolor immunohistochemistry further confirmed increased abundance of LSK, CMP, and GMP populations in the BM of LIP mice compared with that in Ctrl mice (Figure 1J). Overall, these data indicate that local chronic inflammation leads to sustained hematopoietic adaptation in the BM, characterized by increased proliferation and a shift toward myeloid lineage differentiation in HSPCs.

### Periodontitis skews hematopoietic differentiation toward the neutrophil lineage

**Figure 2:**
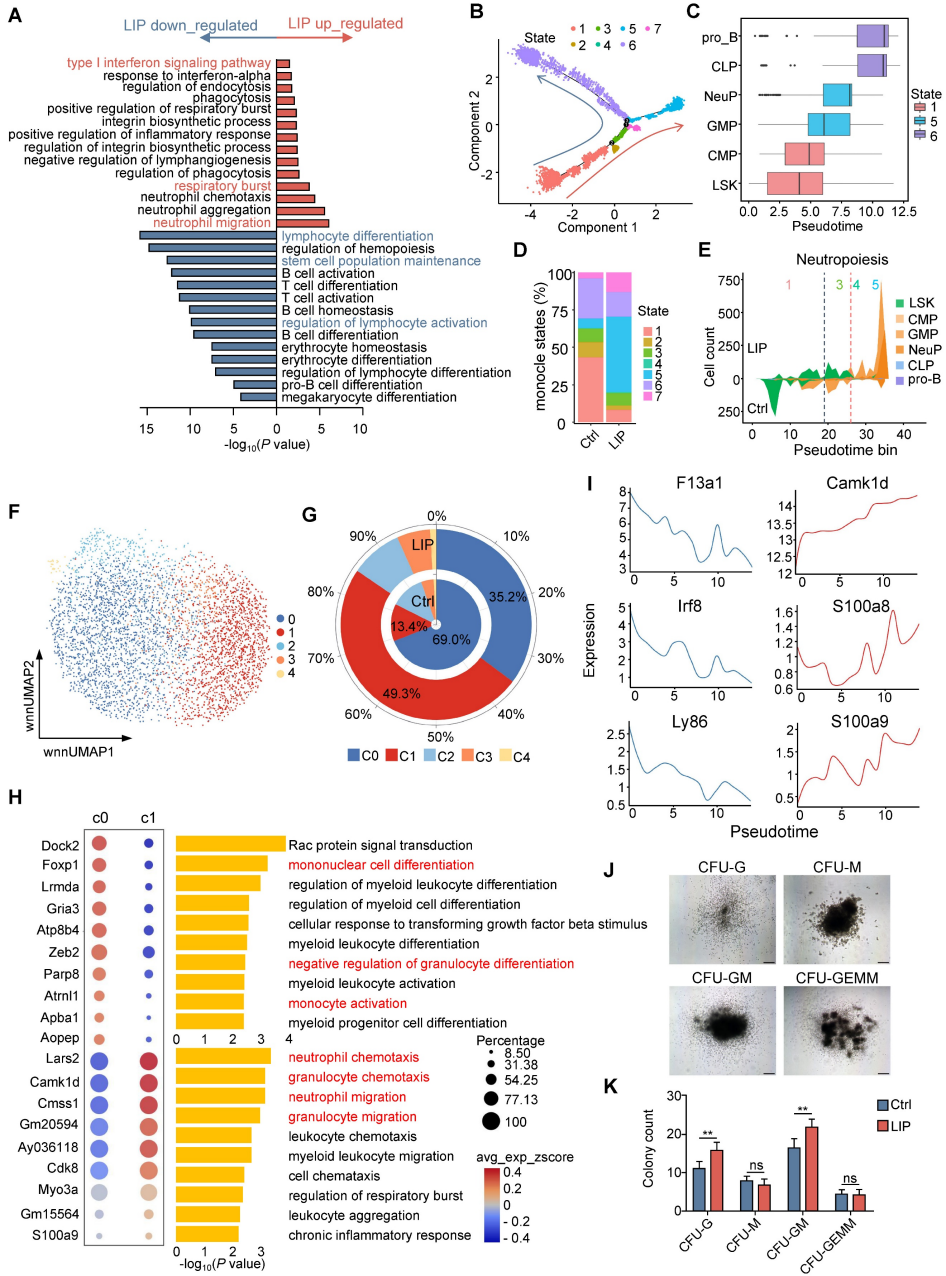
** Periodontitis skews differentiation of HSPCs toward the neutrophil lineage. (A)** Top overrepresented GO:BP terms, including upregulated (red) and downregulated (blue) genes, in LSK cells from LIP mice versus Ctrl mice. **(B)** Differentiation trajectory generated using Monocle 2. Cells are colored according to monocle states. **(C)** Repartition of the Seurat clusters along the pseudotime. Box plots (medians) of pseudotime values are colored according to the most represented state. **(D)** Percentage of monocle states in the Ctrl and LIP groups. **(E)** Stacked plot of predicted cell types along pseudotime cut into 50 bins for LIP (upper part of the plots) and Ctrl (lower part of the plots) for neutropoiesis. Black and red stretched lines mark 2 and 1 bifurcation point pseudotime, respectively. **(F)** Subclustering UMAP plot of all GMPs, colored by cell type. **(G)** Stacked radial chart showing the distribution of GMP subclusters from Ctrl (inner) and LIP (outer) mice. The colors correspond to the identified cell types. **(H)** Bubble plot (left) and GO:BP enrichment analysis (right) of the top 10 marker genes in annotated cell clusters. **(I)** Gene expression along pseudotime of selected markers of monocytopoiesis (left) and neutropoiesis (right). **(J)** Representative hematopoietic colonies from BM cells scored at day 7 of culture. **(K)** Quantitation of colonies formed from BM cells from Ctrl and LIP mice in methylcellulose medium. Data are presented as the mean ± SD from at least three independent experiments; n = 6/group (K). *P* values were calculated using two-tailed Student's *t* test; *P < 0.05, **P < 0.01, ***P < 0.001, ns indicates no significant difference.

Hematopoiesis is a dynamic process governed by transcriptional programs that regulate cell fate decisions 29, 30. To elucidate the underlying mechanisms by which periodontitis drives myeloid bias, transcriptomic analysis was conducted on HSPCs derived from LIP and Ctrl mice. Gene ontology (GO) enrichment analysis of differentially expressed genes (DEGs) in LSK cells revealed significant upregulation of terms associated with “neutrophil migration” and “respiratory burst”, while terms such as “lymphocyte differentiation,” “stem cell population maintenance,” and “regulation of lymphocyte activation” were enriched among downregulated genes (Figure 2A). To gain further insight into the impact of periodontitis on hematopoietic lineage differentiation, differentiation trajectory of representative progenitor populations—including LSK, CMP, GMP, NeuP, CLP, and pro_B—were inferred using scRNA-seq data (Figure S2A). The reconstructed trajectory comprised seven distinct cellular states. State 1, with the highest percentage of LSK cells, was positioned at the initiation of the trajectory, whereas states 5 and 6 dominated both ends of the differentiation trajectory (Figure 2B and Figure S2B). The trajectory predominantly comprised cells from the Ctrl group, with bifurcation into two branches primarily occupied by cells from either the LIP or Ctrl groups (Figure S2C). Mapping of Seurat clusters across the differentiation trajectory reinforced the relevance of pseudotime progression to lineage commitment and highlighted the differentiation specificity of each cellular state (Figure 2C). Notably, States 1 and 6, composed largely of LSK, CLP, and pro_B clusters, were enriched in the Ctrl group. In contrast, state 5—dominated by GMP and NeuP clusters—was expanded in the LIP group (Figure 2D and Figure S2D). Visualization of the distribution of representative progenitor subsets further confirmed that LSK cells in the LIP group preferentially underwent myeloid differentiation, particularly toward the neutrophil lineage, while lymphoid differentiation was more prominent in the Ctrl group (Figure 2E and Figure S2E-G).

Given that GMPs represent the most differentiated myeloid progenitor subset responsible for generating monocytes and neutrophils, the impact of periodontitis on myeloid progenitor cell fate decisions was further explored using an unbiased approach. All GMPs were classified into five subclusters, among which subcluster C0 was primarily enriched in the Ctrl group, while C1 was enriched in the LIP group (Figure 2F-G). GO enrichment analysis of cluster-specific marker genes revealed that C0 was associated with terms such as “mononuclear cell differentiation” and “negative regulation of granulocyte differentiation,” whereas C1 showed enrichment of pathways related to neutrophil activation (Figure 2H). In parallel, C1 demonstrated increased chromatin accessibility at transcription factor (TF) motifs associated with the CEBP family, including *Cebpa*, *Cebpb*, *Cebpd*, and *Cebpe* (Figure S2H), suggesting enhanced susceptibility to neutrophil lineage differentiation. Reconstruction of the lineage trajectory of GMP subclusters based on scRNA-seq data revealed that cells from the Ctrl group localized primarily at the initiation of the trajectory, while those from the LIP group occupied more terminal positions (Figure S2I-K). Periodontitis induced the development of GMPs toward the neutrophil lineage at the expense of monocytic differentiation, as indicated by the dynamic changes in monocytic signature genes (*F13a1*, *Irf8*, and *Ly86*) and neutrophil-associated genes (*Camk1d*, *S100a8*,* and S100a9*) along the trajectory (Figure 2I). Collectively, these data suggest that periodontitis leads to transcriptomic reprogramming of HSPCs, resulting in a skewed differentiation toward neutropoiesis while suppressing lymphopoiesis and monocytopoiesis. To further support these findings, colony-forming unit (CFU) assays were conducted using BM cells from Ctrl and LIP mice. Seven days post-assay, a marked increase in CFU-granulocyte (CFU-G) and CFU-granulocyte and monocyte (CFU-GM) colonies was observed in the LIP group compared to the Ctrl group (Figure 2J-K), providing functional evidence for enhanced granulocytic potential under inflammatory conditions, consistent with a shift toward neutropoiesis bias.

In addition, GO enrichment analysis of significantly upregulated genes in NeuP from LIP mice revealed enrichment in pathways related to “protein folding,” “response to endoplasmic reticulum stress,” and “response to lipopoly-saccharide (Figure S2L). Notably, marker genes associated with neutrophil activation, such as *Camk1d*, *Prtn3*, *Elane*, and *MPO*, were also upregulated in LIP-derived NeuP (Figure S2M). Overall, these findings suggest that NeuP from LIP mice exhibit a hyperinflammatory phenotype, indicating that local chronic inflammation promotes neutrophil priming.

### Visualizing gene regulatory features during periodontitis-induced neutropoiesis bias

**Figure 3:**
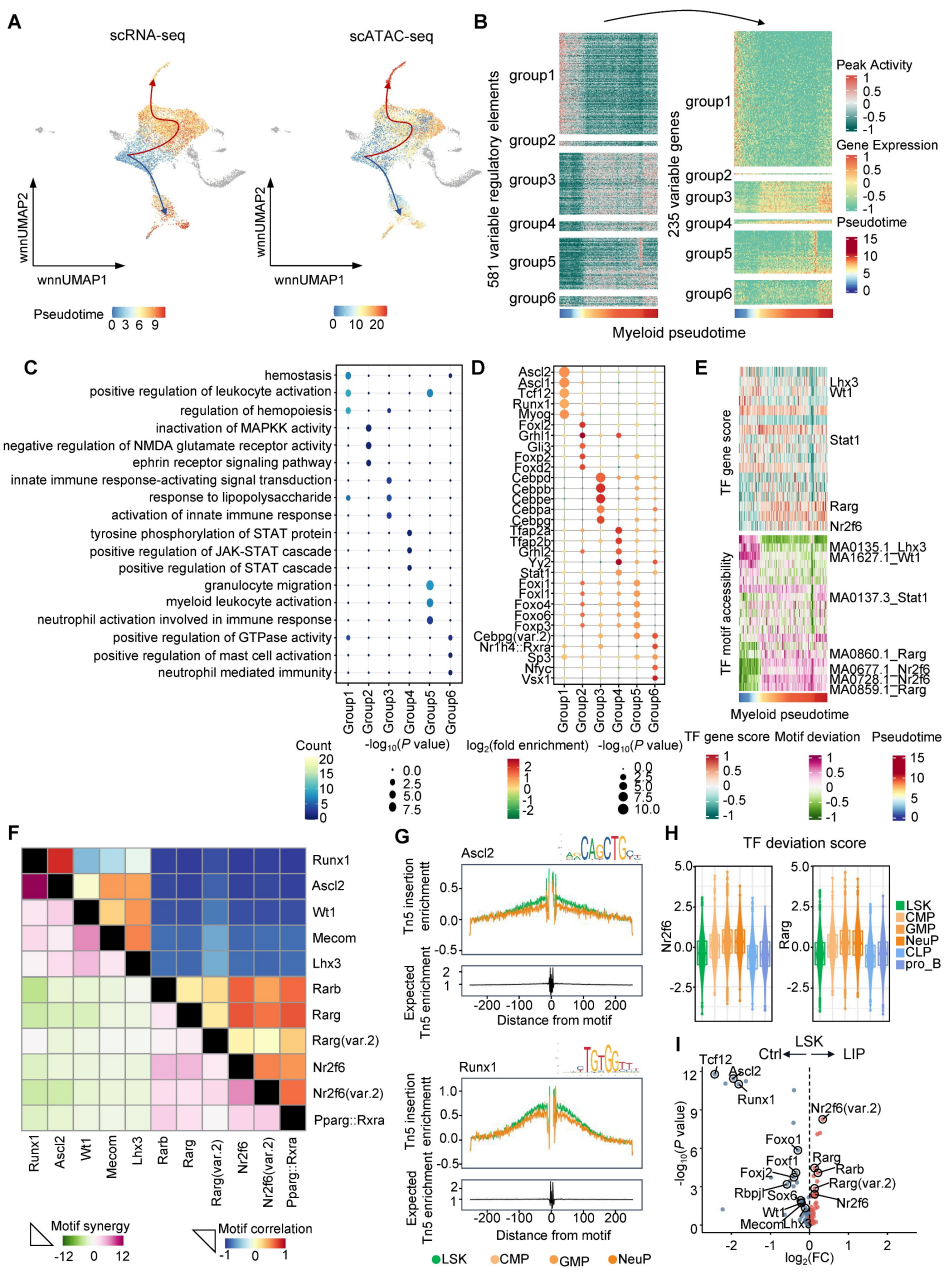
** Dynamic molecular features during periodontitis-induced neutropoiesis bias. (A)** UMAP highlighting the lymphoid and myeloid lineage trajectories based on scRNA-seq data (left) and scATAC-seq data (right). **(B)** Accessibility (left) and expression (right) dynamics across myeloid pseudotime. **(C)** GO enrichment terms associated with genes categorized into six interaction clusters. **(D)** Enrichment analysis of TF motifs within peaks corresponding to these six interaction clusters. Color intensity indicates log_2_(fold enrichment), while point size reflects -log_10_(*P* value). **(E)** Heatmaps showing the positive TF regulators obtained from the integration of ordered TF gene scores (top) with ordered TF motif accessibility (bottom) across myeloid pseudotime. **(F)** TF motif correlation coefficients (upper) and synergy Z scores (lower) of the 11 motifs. **(G)** TF footprints of the Ascl2 (top) and Runx1 (bottom) motif in LSK, CMP, GMP, and NeuP clusters. The Tn5 insertion bias track is shown. **(H)** TF deviation score of *Nr2f6* (left) and *Rarg* (right) in the indicated cell types. **(I)** Volcano plots demonstrating the differential TF motif accessibility using the mean TF motif accessibility in the chromVAR TF bias-corrected deviation between the Ctrl and LIP groups.

To investigate gene regulatory dynamics during periodontitis-driven neutropoiesis bias, tran-scriptomic and chromatin accessibility changes were analyzed along continuous differentiation trajectories. Lineage differentiation trajectories were first constructed using both scRNA-seq and scATAC-seq data, which showed consistent patterns (Figure 3A). To visualize the complete repertoire of dynamic regulatory profiles, regulatory elements were ordered based on their accessibility changes along inferred myeloid and lymphoid trajectories and grouped into six clusters. These dynamic regulatory elements and their associated genes aligned with sequential differentiation states (Figure 3B and Figure S3A). Genes linked to early myeloid pseudotime were enriched in GO terms such as “positive regulation of leukocyte activation” and “regulation of hemopoiesis,” whereas genes activated later along the myeloid trajectory were associated with “granulocyte migration” and “myeloid leukocyte activation” (Figure 3C). In contrast, genes active in the late lymphoid pseudotime were enriched for terms including “B cell proliferation” and “lymphoid progenitor cell differentiation” (Figure S3B). To identify potential TFs governing these regulatory programs, motif enrichment analysis was performed across regulatory element clusters (Figure 3D and Figure S3C). TF motifs enriched during intermediate and late stages of myeloid differentiation included key regulators of neutrophil lineage such as *Cebpa*, *Cebpb*, *Cebpd*, and *Cebpe* (Figure 3D). To further characterize the TF-driven regulatory dynamics of hematopoiesis, TF gene scores were correlated with chromVAR-derived TF deviation scores across pseudotime. Toward the final stage of the lymphoid lineage trajectory from LSK to the pro_B, TF such as *Foxo1*, *Foxj2*, *Tcf12*, and *Rbpjl* were identified as positive regulators of lymphoid differentiation (Figure S3D). Motif accessibility for *Rarg* and *Nr2f6* was elevated during early and intermediate stages of the lymphoid lineage trajectory but decreased upon differentiation into lymphoid progenitors (Figure S3D). Notably, *Rarg* and *Nr2f6* motif accessibility remained high during the transition from LSK to GMP and NeuP, suggesting potential roles in promoting neutropoiesis (Figure 3E). To investigate TF coordination during neutropoiesis, genome-wide synergy and correlation patterns of motif family accessibility were analyzed (Figure 3F). Two broad classes of motifs were identified: (1) early-activity motifs such as *Runx1*, *Ascl2*, *Wt1*, *Mecom*, and *Lhx3*, which exhibited high synergy and correlation and were primarily associated with stem cell maintenance at the onset of neutropoiesis; and (2) late-activity motifs including *Rarb*, *Rarg*, *Nr2f6*, and *Pparg*, which were highly intercorrelated and implicated in skewing hematopoietic lineage differentiation toward neutrophils. These findings highlighted key TFs potentially regulating neutropoiesis during inflammatory hematopoiesis. Increased accessibility of *Ascl2* and *Runx1* motifs were observed in the LSK cluster, supporting their roles in stem cell self-renewal and maintenance (Figure 3G). In parallel, progression along the neutrophil lineage was accompanied by increased accessibility of motifs for *Nr2f6*, *Rarg*, *Rarb*, and *Rxra*, and decreased accessibility of motifs associated with lymphoid regulators such as *Tcf12*,* Lhx3*, *Rbpjl*, *Foxo1*, *Foxj2*, and *Foxf1* (Figure 3H and Figure S3E).

TF deviation scores and variation analyses were subsequently used to identify differentially enriched positive TF regulators in LSK cells from Ctrl and LIP groups. Stemness-associated TFs, including *Ascl2*, *Runx1*, *Sox6*, *Mecom*, *Wt1*, and *Lhx3*, as well as lymphoid differentiation TFs such as *Foxo1*, *Foxf1*, *Foxj2*, *Tcf12*, and *Rbpjl*, were enriched in the Ctrl group. In contrast, *Rarg* and *Nr2f6*, TFs implicated in neutropoiesis, were enriched in the LIP group (Figure 3I). Collectively, integrated pseudotime-based analysis of TF activity identified distinct sets of positive regulators that support HSC stemness and lymphoid lineage differentiation under homeostatic conditions, while facilitating neutropoiesis during local chronic inflammation. Among these, *Rarg* and *Nr2f6* were identified as the most likely regulators mediating the neutropoiesis bias induced by local chronic inflammation.

### Periodontitis induces neutrophil priming

**Figure 4:**
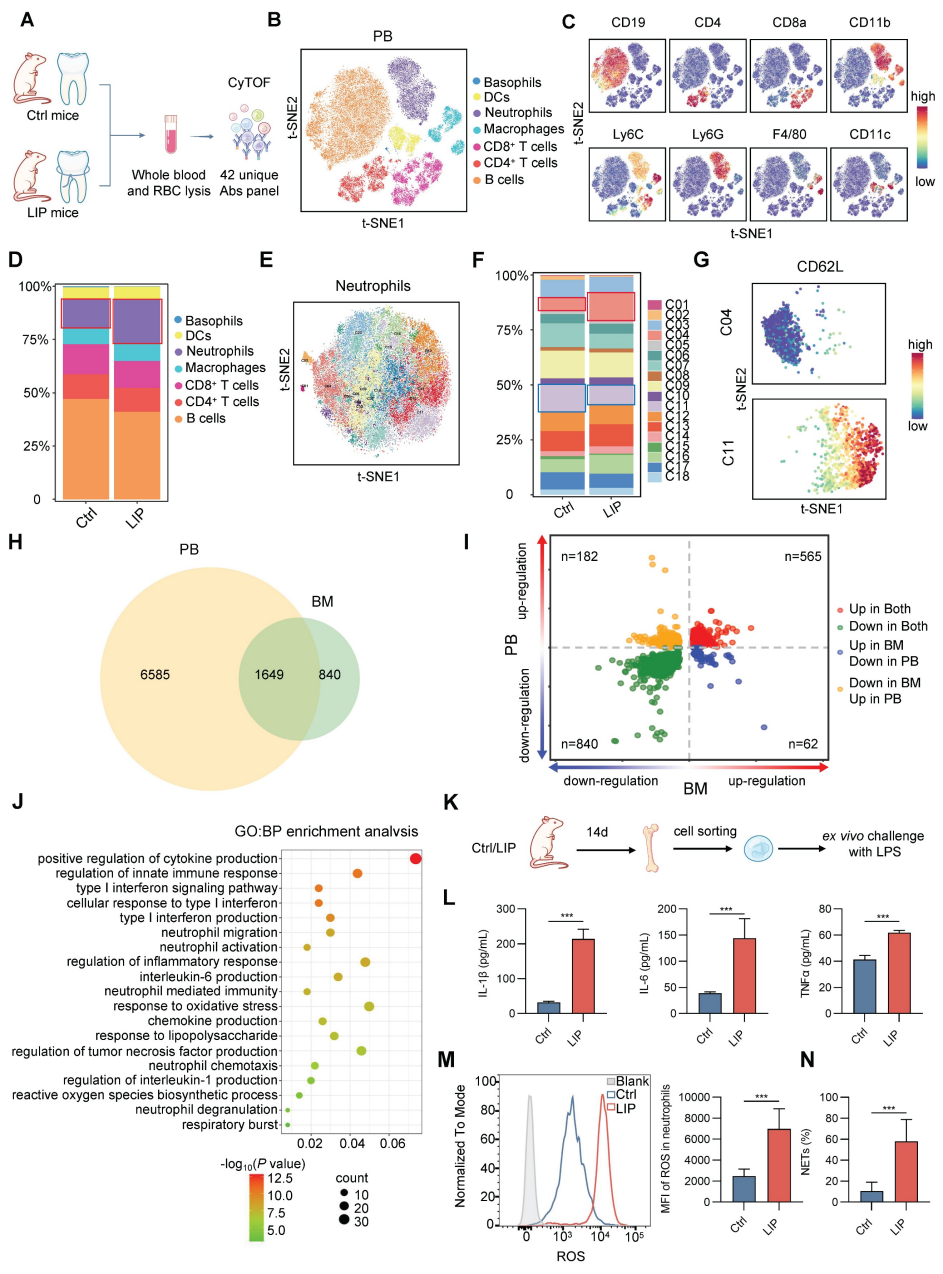
** Periodontitis induces neutrophil priming. (A)** Schematic for CyTOF of leukocytes in whole-blood samples. **(B)** Visualized t-SNE maps of CD45^+^ leukocytes from PB; maps were based on the expression of 42 different markers. **(C)** Expression levels of representative cell markers in the resulting t-SNE clustered cell populations are shown. **(D)** Bar chart of the relative frequency of CD45^+^ leukocytes in the PB of Ctrl and LIP mice. **(E)** Visualized t-SNE maps of neutrophil subclusters from PB; maps were based on the expression of 42 different markers. **(F)** Bar chart of the relative frequency of neutrophil subclusters in the PB of Ctrl and LIP mice. **(G)** Visualized t-SNE maps of C04 and C11, colored according to expression intensity of CD62L. **(H)** Comparison of Smart-seq2 data from PB and RNA-seq from BM in Ctrl and LIP mice. Venn diagram depicting an overlap of 1649 genes between periodontitis-induced differential gene expression of PB and BM neutrophils. DEGs are defined by *P* < 0.05. **(I)** Two-way volcano plot depicting overlap between PB DEGs (y axis) and BM DEGs (x axis); red dots indicate upregulated genes (n = 565) and green dots indicate downregulated genes (n = 840) in both datasets. **(J)** GO:BP enrichment analysis for 565 DEGs commonly upregulated in both PB and BM neutrophils. **(K)** Experimental design for secondary challenge model. **(L)** LPS-stimulated cytokine responses in neutrophils isolated from BM of Ctrl and LIP mice. **(M)** Overlay histogram of ROS production in neutrophils (left) and the corresponding mean fluorescence intensity (right). **(N)** Quantitative analysis of NETs formation in neutrophils. Data are presented as the mean ± SD from at least three independent experiments; n = 6/group (L-N). *P* values were calculated using two-tailed Student's *t* test; *P < 0.05, **P < 0.01, ***P < 0.001, ns indicates no significant difference.

Flow cytometry was used to evaluate the impact of periodontitis on BM immune cell composition. A marked increase in neutrophil frequency was observed, accompanied by a reduction in lymphocyte populations, including B cells, CD4^+^ T cells, and CD8^+^ T cells (Figure S4A). Subsequently, alterations in major leukocyte lineages were characterized in peripheral blood (PB) samples from Ctrl and LIP mice after 14 d of silk ligation, employing mass cytometry (CyTOF) to profile surface marker expression (Figure 4A-C). Consistent with BM findings, a higher proportion of neutrophils and reduced lymphocyte frequencies were observed in LIP mice compared to Ctrl mice (Figure 4D). These results were further validated by flow cytometric analysis of PB immune cell composition (Figure S4B). Unsupervised cluster analysis of PB neutrophils revealed a markedly higher proportion of Cluster 04 (CD62L^-^ neutrophils) and a lower proportion of Cluster 11 (CD62L^+^ neutrophils) in LIP mice relative to Ctrl mice (Figure 4E-G and Figure S4C). To further characterize the activation status of circulating neutrophils, expression levels of established surface markers, including CD11b, CD66a, and CD62L, were assessed. Neutrophils from LIP mice exhibited significantly increased expression of CD11b and CD66a, along with reduced CD62L expression (Figure S4D), indicating a primed activation state in the context of periodontitis. To further investigate neutrophil priming, transcriptomic integration of Smart-seq2 data from PB neutrophils and RNA-seq data from BM neutrophils identified 565 DEGs that were commonly upregulated (Figure 4H-I). GO enrichment analysis of these DEGs revealed significant upregulation of pathways associated with reactive oxygen species (ROS) production, chemotaxis, cytokine and chemokine production, degranulation, and respiratory burst, suggesting transcriptional reprogramming of neutrophils toward a pro-inflammatory phenotype (Figure 4J). Functional assays confirmed this shift, as neutrophils from LIP mice displayed increased production of pro-inflammatory cytokines (IL-1β, IL-6, and TNF-α), elevated ROS levels, and enhanced neutrophil extracellular trap (NET) formation following lipopolysaccharide (LPS) stimulation, compared to neutrophils from Ctrl mice (Figure 4K-N). Collectively, these findings suggest that local chronic inflammation associated with periodontitis promotes expansion of the neutrophil population in both BM and peripheral circulation and primes these cells toward a pro-inflammatory phenotype, thereby enhancing responsiveness to systemic inflammatory stimuli.

### Periodontitis-induced neutrophil priming contributes to periodontitis-arthritis comorbidity

**Figure 5:**
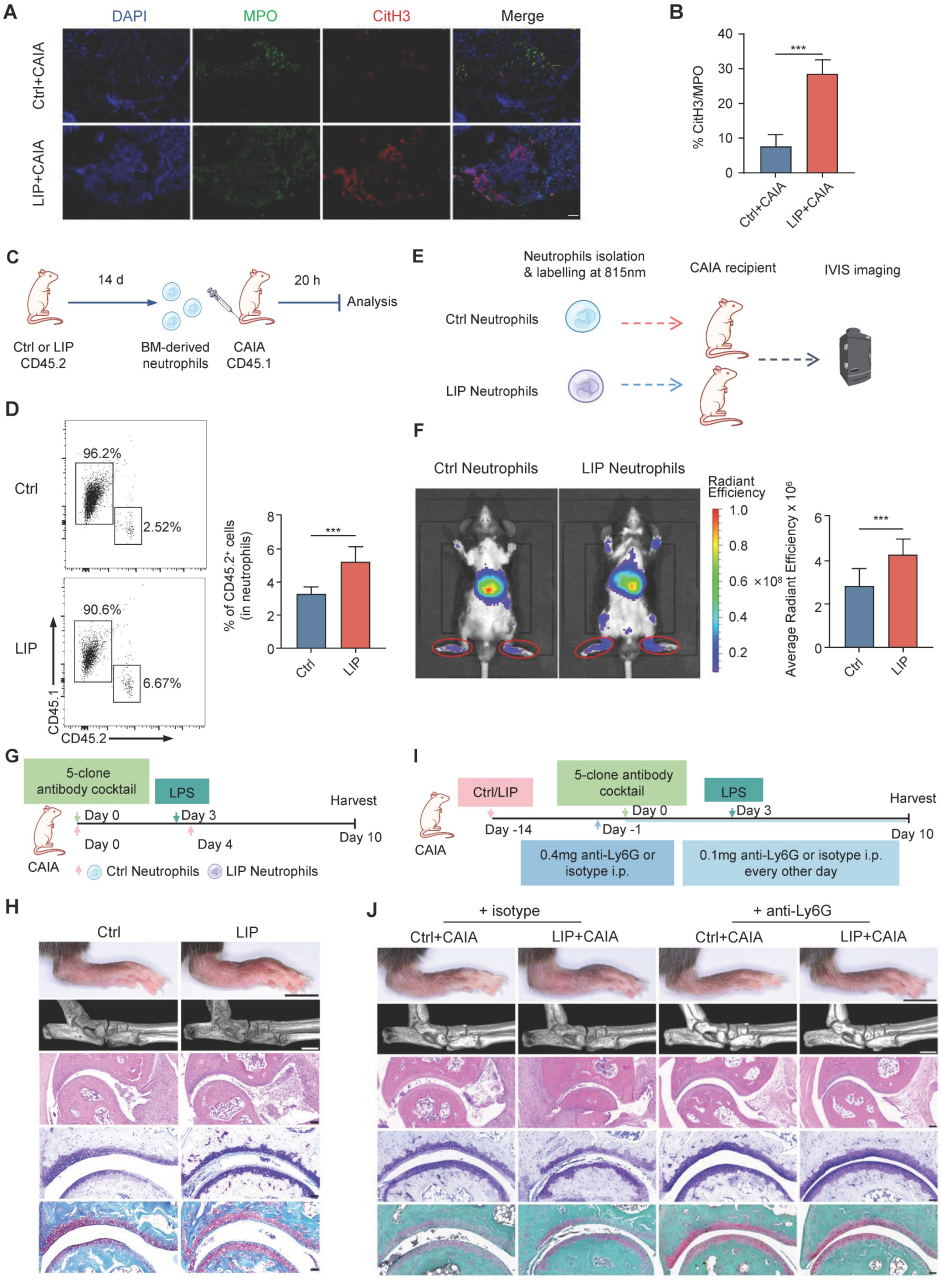
** Periodontitis-induced neutrophil priming underlies the periodontitis-arthritis comorbidity. (A)** Representative images of ankle joint sections stained with citH3 (red), MPO (green), and DAPI (blue). NETs are visualized via colocalization of citH3 and DAPI staining (merged images) (scale bar = 50 µm). **(B)** Percentage of NETs area in the synovium of the ankle joints normalized to an MPO-positive signal. **(C)** Schematic diagram showing the experimental design for neutrophil adoptive transfers. **(D)** Representative FACS plots to identify CD45.2^+^ cells and CD45.1^+^ cells in neutrophils in the synovium of joints (left). Quantification of percentages of CD45.2^+^ cells in neutrophils in the synovium of joints (right). **(E)** Schematic showing the neutrophil tracing experimental design. **(F)** Whole body IVIS fluorescence images of 815 nm dye-labeled Ctrl and LIP neutrophil tracking to the ankle joints of CAIA recipient mice. Graph showing average radiant efficiency of IVIS imaged mice (left). Quantitative analyses of average radiant efficiency of the hind paws of IVIS-imaged mice (right). **(G)** Schematic of experimental design for the adoptive transfer of neutrophils during CAIA model induction. **(H)** Representative photographs of hindlimbs at the endpoint of the experiment from Ctrl and LIP groups (scale bar = 5 mm); representative micro-CT images of ankle joints (scale bar = 1 mm); histopathological evaluation of ankle joints was performed using H&E (scale bar = 100 µm), toluidine blue, and safranin-O/fast green staining (scale bar = 50 µm). **(I)** Schematic of experimental design for Ly6G blockade during CAIA model induction. **(J)** Representative photographs of hindlimbs at the endpoint of the experiment from different treatment groups (scale bar = 5 mm); representative micro-CT images of ankle joints (scale bar = 1 mm); histopathological evaluation of ankle joints was performed using H&E (scale bar = 100 µm), toluidine blue, and safranin-O/fast green staining (scale bar = 50 µm). Data are presented as the mean ± SD from at least three independent experiments; n = 8 mice/group (B, D and F). *P* values were calculated using two-tailed Student's* t* test; *P < 0.05, **P < 0.01, ***P < 0.001, ns indicates no significant difference.

Consistent with epidemiological studies 9, 31, mice subjected to LIP exhibited significantly more severe arthritic damage than Ctrl mice when assessed using the collagen antibody-induced arthritis (CAIA) model, which recapitulates key features of human arthritis 32 (Figure S5A-F). Given the established role of dysregulated neutrophil activation in arthritis pathogenesis 33, 34, the contribution of neutrophil priming to periodontitis-exacerbated arthritis was examined. Histological analysis of ankle joint lesions demonstrated a higher infiltration of neutrophils, indicated by myeloperoxidase-positive (MPO^+^) staining, in LIP + CAIA mice compared to Ctrl + CAIA mice (Figure 5A). NETs were abundant in the ankle joint sections from LIP + CAIA mice but were scarcely detected in Ctrl + CAIA mice, as quantified by the percentage of citrullinated histone H3 (citH3)-positive areas within MPO^+^ regions (Figure 5B). An *in vitro* chemotaxis assay demonstrated that neutrophils isolated from LIP mice exhibited enhanced migratory activity relative to those from Ctrl mice (Figure S5G-H). To directly assess the homing capacity of neutrophils from Ctrl and LIP mice to inflamed ankle joints in the CAIA model, adoptive transfer experiments were performed. Equal numbers of neutrophils isolated from CD45.2 Ctrl or LIP mice were intravenously administered into CD45.1 CAIA recipient mice (Figure 5C). Flow cytometric analysis showed increased accumulation of donor-derived neutrophils in the synovium of mice that received LIP-derived neutrophils, indicating enhanced homing capacity (Figure 5D). For *in vivo* tracking, fluorescently labeled neutrophils were intravenously administered to CAIA recipient mice. Imaging performed 20 h post-injection fluorescence imaging revealed significantly stronger fluorescence signals in mice receiving LIP-derived neutrophil, suggesting enhanced homing to inflamed joints (Figure 5E-F). Notably, the transfer of primed neutrophils from LIP mice led to a pronounced exacerbation of arthritis symptoms in recipient mice, compared to those receiving neutrophils from Ctrl mice (Figure 5G-H and Figure S5I-L). Functional blockade of neutrophils using anti-Ly6G monoclonal antibodies (mAbs) in the CAIA model (Figure 5I) effectively abrogated the pro-inflammatory effects associated with periodontitis. LIP mice no longer exhibit aggravated arthritis severity following neutrophil depletion, indicating that neutrophil activity is essential for mediating this comorbidity (Figure 5J and Figure S5M-P). These findings demonstrate that local inflammation-induced neutrophil priming plays a key role in the development of inflammatory comorbidities, including arthritis.

### IFN-I mediates neutropoiesis bias and neutrophil priming in periodontitis

**Figure 6:**
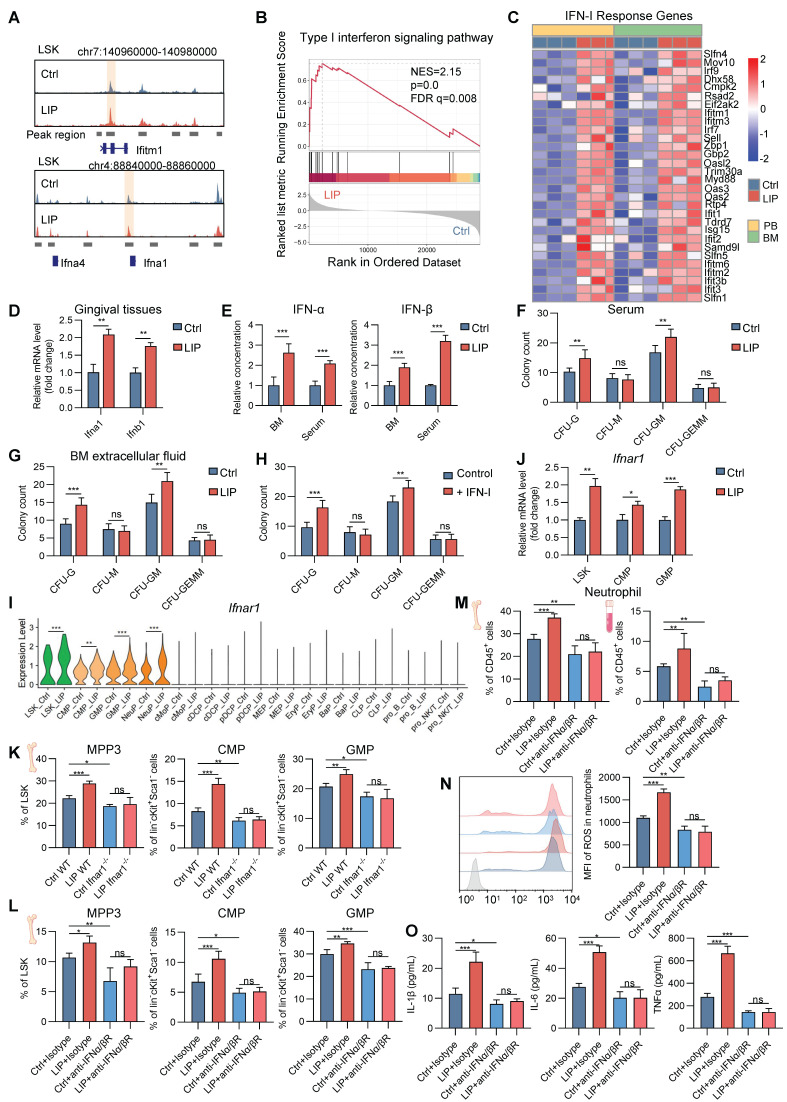
** IFN-I drives neutropoiesis bias and neutrophil priming in periodontitis. (A)** Genome browser track showing DARs in the *Ifitm1* locus and proximity to the *Ifna1* locus. **(B)** GSEA for genes related to the IFN-I signaling pathway in neutrophils from LIP mice compared with neutrophils from Ctrl mice. **(C)** Heatmap showing relative expression of genes in the cell response to IFN-I gene set between neutrophils isolated from PB or BM. **(D)** RT-qPCR analysis of *Ifna1* and *Ifnb1* mRNA expression in gingival tissues from Ctrl and LIP mice. **(E)** Quantification of IFNα (left) and IFNβ (right) levels in BM extracellular fluid and serum from LIP mice, normalized to those from Ctrl mice. **(F-G)** CFU assays of BM cells from WT mice cultured in medium containing serum **(F)** or BM extracellular fluid **(G)** from either Ctrl or LIP mice. Colony numbers were quantified after 7 d of culture. **(H)** Quantification of colony formation from BM cells from WT mice cultured in medium containing either IFN-I-free or IFN-I-containing medium. **(I)** Violin plots showing the expression levels of *Ifnar1* in various HSPC populations from Ctrl and LIP mice. **(J)** RT-qPCR analysis of *Ifnar1* mRNA expression in LSK, CMP, and GMP from Ctrl and LIP mice. **(K-L)** Frequencies of MPP3 in LSK cells. Frequencies of CMP and GMP in Lin^-^cKit^+^Sca1^-^ cells. **(M)** Frequencies of CD11b^+^Ly6G^+^ neutrophils in CD45^+^ cells in the BM and PB of mice. **(N)** Overlay histogram of ROS production in neutrophils (left) and the corresponding mean fluorescence intensity (right). **(O)** BM neutrophils, isolated from mice treated as indicated, were stimulated with LPS. The concentrations of IL-1β, IL-6, and TNF-α in the supernatant are shown. Data are presented as the mean ± SD from at least three independent experiments; n = 6 mice/group **(D-H, J-O)**. *P* values were calculated using two-tailed Student's *t* test **(D-H, J)** and one-way ANOVA **(K-O)**; *P < 0.05, **P < 0.01, ***P < 0.001, ns indicates no significant difference.

To explore the intricate mechanisms by which periodontitis drives neutropoiesis bias, GO enrichment analysis was performed on DEGs in LSK cells, NeuPs, and neutrophils. Significant enrichment of genes associated with the IFN-I signaling pathway was observed in the LIP group (Figure 2A, Figure S2L, and Figure 4J). During the differentiation transition from LSK cells to GMP and NeuP, enrichment of JAK/STAT signaling-related terms was detected, along with increased chromatin accessibility at the *Stat1* motif, suggesting activation of IFN-I signaling pathway-associated transcriptional programs (Figure 3C-E). Genome browser track analysis revealed increased chromatin accessibility at the *Ifitm1* locus and near the promoter region of the *Ifna1* gene in LSK cells from LIP mice compared to Ctrl mice (Figure 6A). Gene set enrichment analysis (GSEA) further demonstrated positive correlation with the IFN-I signaling pathway in neutrophils from LIP mice compared to those from Ctrl mice (Figure 6B), consistent with the upregulation of IFN-I response genes in LIP-derived neutrophils (Figure 6C). Elevated expression of *Ifna1* and *Ifnb1* was detected in gingival tissues of LIP mice relative to Ctrl mice (Figure 6D), and correspondingly, significantly higher levels of IFNα and IFNβ were found in both BM extracellular fluid and serum in the LIP mice compared to Ctrl group (Figure 6E). Collectively, these findings suggest that IFN-I produced locally in gingival tissues may enter the systemic circulation and act on the BM to promote neutropoiesis bias through activation of the IFN-I signaling pathway.

To functionally validate the role of IFN-I, CFU assays were performed using BM cells from wild-type (WT) mice cultured in media supplemented with serum or BM extracellular fluid from either Ctrl or LIP mice. After 7 d, cultures supplemented with LIP-derived serum or BM extracellular fluid exhibited a marked increase in CFU-G and CFU-GM colonies compared to controls (Figure 6F-G). In a parallel experiment, CFU assays performed in IFN-I-free or IFN-I-supplemented media revealed a significant increase in CFU-G and CFU-GM colonies in the IFN-I treatment group compared to controls (Figure 6H), supporting a direct role for IFN-I in mediating neutropoiesis bias in the context of periodontitis.

Next, to identify the target cells of IFN-I in the BM, expression of the IFN-I receptor subunit *Ifnar1* was examined. Significantly elevated *Ifnar1* expression was observed in LSK, CMP, GMP, and NeuP populations from LIP mice compared to Ctrl mice (Figure 6I-J), suggesting that IFN-I may act directly on these cell populations by activating IFN-I signaling in these cells to induce skewed neutropoiesis in periodontitis. To functionally link IFN-I signaling to LIP-induced skewed neutropoiesis, Ifnar1-deficient (Ifnar1^-/-^) mice, which lack the IFN-I receptor, were utilized. In these mice, LIP-induced neutropoiesis bias was abolished, as evidenced by the absence of significant differences in the frequencies of myeloid-biased MPP3, CMP, and GMP populations between Ctrl and LIP groups, in contrast to the skewing observed in WT mice (Figure 6K). Similarly, treatment of Ctrl and LIP mice with a neutralizing antibody targeting the IFNα/β (IFNα/βR) receptor abrogated the LIP-induced skewed neutropoiesis (Figure 6L). Moreover, IFNα/βR blockade in LIP mice reversed the increase in neutrophil abundance in both BM and PB observed in untreated LIP mice (Figure 6M). Neutralization of IFNα/βR also suppressed the pro-inflammatory phenotype of LIP-primed neutrophils, as reflected by reduced ROS production and diminished release of pro-inflammatory cytokines upon LPS stimulation (Figure 6N-O). Together, these results establish a critical role for IFN-I signaling in regulating neutropoiesis bias and neutrophil priming in the setting of periodontitis.

To investigate whether inhibition of IFN-I signaling ameliorates arthritis symptoms in the context of periodontitis, arthritis was induced in Ctrl and LIP mice treated with anti-IFNα/βR mAb or isotype control. Anti-IFNα/βR mAb treatment eliminated periodontitis-associated arthritis exacerbation, as evidenced by comparable levels of hind paw thickness, bone erosion, synovial inflammation, and cartilage destruction between Ctrl + CAIA and LIP + CAIA groups receiving anti-IFNα/βR mAb (Figure S6A-E). To further explore the mechanism by which IFN-I blockade alleviates periodontitis-aggravated arthritis, CAIA mice that had previously received adoptive transfer of periodontitis-primed neutrophils were treated with either anti-IFNα/βR or isotype control (Figure S6F). Mice receiving anti-IFNα/βR mAb exhibited significantly improved arthritis outcomes compared to the isotype control group (Figure S6G-J). These findings suggest that the therapeutic potential of IFN-I signal inhibition in periodontitis-aggravated arthritis is predominantly mediated through the modulation of neutropoiesis bias and neutrophil priming.

Additionally, TF regulatory network analysis revealed that *Rarg* and *Nr2f6* are linked to IFN-I signaling-associated genes (Figure S6K). Supporting their regulatory potential, genome browser track analysis showed increased chromatin accessibility at the *Stat1* locus in LSK cells from LIP mice (Figure S6L). Furthermore, motif scanning using TFBStools identified high-confidence *Rarg* and *Nr2f6* binding sites within the *Stat1* promoter region (homology >85%, binding score >8; Table S1). These results suggest that *Rarg* and *Nr2f6* may directly regulate *Stat1* expression and functionally contribute to IFN-I signaling-driven neutropoiesis in the context of periodontitis.

### Periodontal inflammation resolution alleviates arthritis progression by reversing neutropoiesis bias

**Figure 7:**
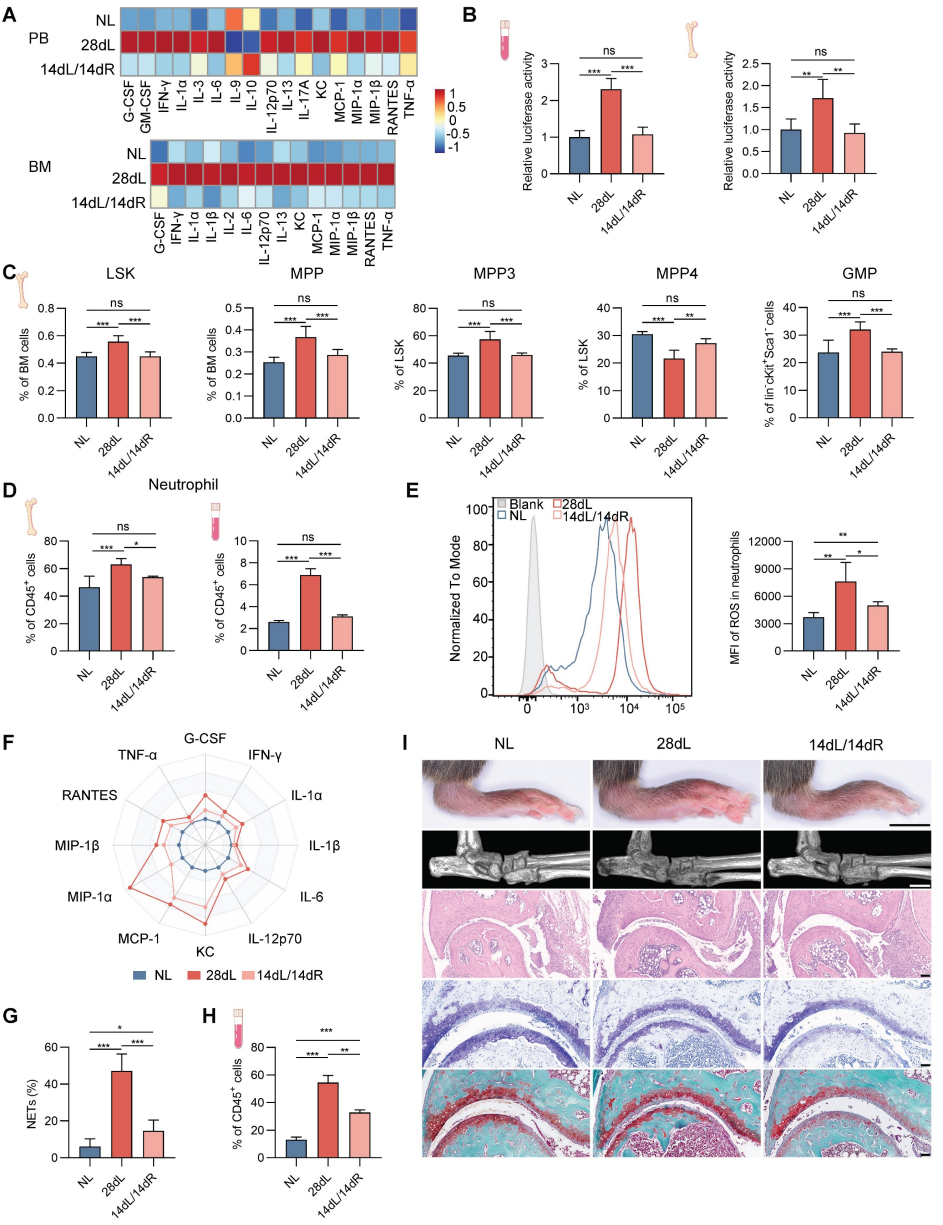
** Periodontitis resolution alleviates the progression of periodontitis-related arthritis by reversing neutropoiesis bias. (A)** Heatmap representing normalized serum (top) and BM extracellular fluid (bottom) cytokine levels from NL, 28dL, and 14dL/14dR mice. **(B)** The levels of active IFN-I in serum and BM extracellular fluid were measured using the IFN bioassay. **(C)** Frequencies of LSK and MPP in total BM cells. Frequencies of MPP subsets (MPP3 and MPP4) in LSK cells. Frequencies of GMP in Lin^-^cKit^+^Sca1^-^ cells. **(D)** Frequencies of CD11b^+^Ly6G^+^ neutrophils in CD45^+^ cells in the BM (left) and PB (right) of mice. **(E)** Overlay histogram of ROS production in neutrophils (left) and the corresponding mean fluorescence intensity (right), following *in vitro* LPS stimulation. **(F)** BM neutrophils, isolated from mice treated as indicated, were stimulated with LPS (100 ng/mL) *in vitro* for 17 h. Radar plots illustrate the normalized concentrations of cytokines in the supernatant. **(G)** Quantitative analysis of NETs formation in neutrophils, following *in vitro* LPS stimulation. **(H)** Frequencies of neutrophils in CD45^+^ cells in the PB of mice restimulated with LPS. **(I)** Representative photographs of hindlimbs at the endpoint of the experiment from NL, 28dL, and 14dL/14dR groups (scale bar = 5 mm); representative micro-CT images of ankle joints (scale bar = 1 mm); histopathological evaluation of ankle joints was performed using H&E (scale bar = 100 µm), toluidine blue, and safranin-O/fast green staining (scale bar = 50 µm). Data are presented as the mean ± SD from at least three independent experiments; n = 6 mice/group **(B-E, G, H)** and n = 3/group **(F)**. *P* values were calculated using one-way ANOVA; *P < 0.05, **P < 0.01, ***P < 0.001, ns indicates no significant difference.

To investigate the impact of resolving local chronic inflammation on hematopoietic inflammatory adaptation and associated comorbidities, a comprehensive evaluation was conducted in mice subjected to three conditions: 14 d of LIP followed by 14 d of resolution via ligature removal (14dL/14dR), 28 d of continuous LIP (28dL), and naive controls rested for 28 d (NL). Levels of growth factors (G-CSF), inflammatory cytokines (IFN-γ, IL-1α, IL-6, IL-12p70, IL-13, and TNF-α), and chemokines (KC, MCP-1, MIP-1α, MIP-1β, and RANTES) in BM extracellular fluid and serum, which were elevated after 28 d of LIP exposure, returned to baseline following resolution of periodontitis (Figure 7A). A similar normalization trend was observed for IFN-I levels (Figure 7B). Analysis of BM hematopoiesis revealed that hematopoietic activation and skewed neutropoiesis induced by periodontitis were no longer detectable in mice undergoing resolution. In particular, no significant differences were observed in the proportions of HSPC subsets between the 14dL/14dR and NL groups (Figure 7C and Figure S7A). Similarly, neutrophil counts in BM and PB were not significantly different between 14dL/14dR and NL mice (Figure 7D), indicating that resolution of periodontal inflammation quantitatively restores neutropoiesis to homeostatic levels.

Next, the persistence of periodontitis-induced neutrophil reprogramming following inflammation resolution was investigated. After ligature removal, the capacity of neutrophils to generate ROS, release pro-inflammatory mediators, and form NETs in response to LPS stimulation was significantly reduced in 14dL/14dR mice compared to the 28dL group, although responses remained heightened relative to the NL group (Figure 7E-G and Figure S7B). The *in vivo* response to LPS was further examined, revealing that 72 h after a single intraperitoneal injection of LPS, the frequency of CD11b^+^Ly6G^+^ neutrophils in the PB of 14dL/14dR mice was significantly lower than that of 28dL mice and significantly higher than that of NL mice (Figure 7H). In the context of CAIA, 28dL mice exhibited more severe arthritis compared to both NL and 14dL/14dR mice, as indicated by significantly increased paw thickness and aggravated histopathological features in the ankle joints. However, 14dL/14dR mice also showed greater susceptibility to CAIA than NL mice (Figure 7I and Figure S7C). Altogether, these data strongly support that while resolution of local periodontal inflammation reverses neutropoiesis bias and partially restores neutrophil function, it does not fully eliminate the effects of innate immune training.

## Discussion

This study demonstrates that IFN-I-mediated neutropoiesis bias underlies the development and progression of inflammatory comorbidities. Using the periodontitis-arthritis axis as a model, elevated IFN-I levels induced by experimental periodontitis were shown to drive transcriptional and epigenetic reprogramming of HSPCs, promoting their inflammatory adaptation. These inflammatory adaptations skew hematopoietic differentiation toward the neutrophil lineage, resulting in increased production of primed neutrophils that contribute to the aggravation of comorbid inflammatory conditions, including arthritis. Furthermore, resolution of local inflammation effectively reverses these transcriptional and functional alterations in HSPCs, inhibiting neutrophil lineage skewing and thereby alleviating the associated inflammatory burden.

Hematopoiesis—the dynamic process by which HSPCs generate blood cell lineages—is susceptible to disruption by inflammatory stimuli 16, 18, 19, 35. Through single-cell multiomics sequencing, this study characterized HSPC heterogeneity and delineated hematopoietic lineage differentiation trajectories in response to periodontitis. While previous single-cell investigations of inflammation-associated hemato-poiesis have predominantly focused on LSK or GMP cell populations 15, 36, 37, yielding limited insight into the complex landscape of hematopoiesis, the present study was designed to address this knowledge gap. By profiling a broader population of lin^-^ckit^+^ cells, encompassing both LSK cells and downstream progenitors, sixteen distinct HSPC clusters were identified, demonstrating that periodontitis skews hematopoiesis toward myelopoiesis while reducing lymphopoiesis. Additionally, pseudotime analysis of GMP subclusters suggested that periodontitis induces a lineage bias within GMPs, favoring neutrophil over monocyte differentiation. These findings provide a high-resolution transcriptional and epigenetic landscape of inflammation-regulated hematopoiesis, highlighting how local chronic inflammation alters the BM niche and contributes to systemic immune dysregulation.

Although neutrophils are released from the BM at a steady rate under homeostatic conditions, the mechanisms by which HSPCs direct lineage fate toward neutropoiesis remain elusive 38. Master TFs serve as critical regulators of cell-fate reprogramming 39. In the present study, intrinsic gene regulatory signatures governing hematopoietic lineage differentiation were deciphered using single-cell multiomics profiling. By examining the trajectories of neutrophil production, a set of potential lineage-specific TFs involved in the regulation of neutropoiesis was identified, including *Rarb*, *Rarg*, *Nr2f6*, and *Pparg*. Previous research has predominantly focused on TFs, such as PU.1, GFI1, C/EBPα, and C/EBPε 40-44, which are associated with steady-state neutropoiesis; thus, knowledge of additional regulatory TFs remains limited. These findings may provide new insight into pioneering drivers of cell fate transitions that orchestrate neutrophil lineage specification. Furthermore, comparison of TF deviation scores in LSK cells from periodontitis and control mice revealed that periodontitis may facilitate neutropoiesis by increasing the accessibility of *Rarg* and *Nr2f6*. These observations highlight regulatory mechanisms underlying the skewed differentiation of HSPCs toward the neutrophil lineage in response to inflammation.

HSPCs possess the ability to sense systemic inflammatory signals and respond through increased proliferation and lineage skewing 16, 18, 19, 35. Consistent with existing literature 45-47, periodontitis was found to elevate levels of various growth factors and inflammatory mediators, including G-CSF, IFN-γ, IFN-I, IL-1α, IL-6, and TNF-α. In the context of chronic disorders such as asthma 48 and chronic myelogenous leukemia 49, mediators like G-CSF and IL-6 have been previously identified as disruptors of the hematopoietic hierarchy. Pertaining to periodontitis, IL-1 has been reported to promote trained myelopoiesis 15. Building on these findings, the present study identifies IFN-I as a key contributor to neutropoiesis bias during periodontitis. Despite the absence of exogenous pathogens in the LIP model, IFN-I levels were significantly elevated in gingival tissues, serum, and BM extracellular fluid. Previous studies have demonstrated that tissue damage and cell death can trigger the release of damage-associated molecular patterns, such as mitochondrial DNA, which activate the cGAS-STING pathway in DCs and macrophages, leading to IFN-I production 50, 51. Given the extensive tissue damage and cell death occurring in inflamed gingival tissues of LIP mice, it is plausible that IFN-I is induced through this mechanism. Once released, IFN-I may enter the circulation and reach the BM, where activation of IFN-I signaling in HSPCs ultimately promotes neutrophil lineage skewing. Nevertheless, additional studies are required to fully elucidate the cellular sources and upstream triggers of IFN-I in periodontitis.

IFN-I signaling plays a central role in the pathogenesis of inflammatory and autoimmune diseases such as systemic lupus erythematosus and rheumatoid arthritis, primarily through the regulation of immune cell activation, cytokine production, and antigen presentation at sites of inflammation 52. However, the potential contribution of IFN-I signaling to the systemic dissemination of local inflammation and the development of distant inflammatory comorbidities—particularly through modulation of central immune processes such as hematopoiesis—remains largely unexplored. The present study addresses this gap by demonstrating that local periodontal inflammation activates IFN-I signaling in the BM, resulting in hematopoietic differentiation skewing toward the neutrophil lineage. These neutrophils exhibit heightened inflammatory properties and exacerbate distal comorbid arthritis. This establishes a feed-forward loop in which IFN-I signaling not only modulates mature immune cell function but also reprograms HSPCs, thereby amplifying the systemic consequences of localized inflammation. These findings extend the functional scope of IFN-I signaling and provide novel mechanistic insight into the contribution of chronic oral inflammation to the progression of distant inflammatory comorbidities.

Clinical evidence has demonstrated that white blood cell count is longitudinally associated with periodontitis severity in a dose-dependent manner 53. Furthermore, circulating neutrophils from patients with periodontitis show elevated production of pro-inflammatory mediators following inflammatory stimulation, compared to neutrophils from healthy individuals 23, 54, 55. However, the precise role of neutrophil priming in mediating distal organ damage remains to be fully elucidated. The present findings demonstrate that periodontitis-primed neutrophils exhibit enhanced homing capacity to inflamed arthritic joints and contribute to exacerbated disease severity. Notably, functional blockade of neutrophils using Ly6G-specific antibodies effectively mitigated the aggravating effects of periodontitis on arthritis. These observations suggest that circulating primed neutrophils serve as a mechanistic link between local periodontal inflammation and distant organ comorbidities. Beyond arthritis, excessive recruitment and hyperreactivity of neutrophils have also been implicated in the pathogenesis of other chronic diseases, such as atherosclerosis, diabetes, and non-alcoholic fatty liver disease 56-58. Thus, neutropoiesis bias may represent a common mechanistic pathway through which local chronic inflammation drives the progression of distal organ comorbidities.

Local chronic inflammation represents a significant risk factor for the development and progression of distal organ comorbidities. Li et al. reported that periodontitis may exacerbate symptoms of comorbidities through maladaptive BM-mediated trained innate immunity, with a focus on trained myelopoiesis 15. In contrast to myelopoiesis described in previous studies 15, 36, the present findings demonstrate that periodontitis does not uniformly enhance all myeloid cell output, but instead selectively skews hematopoiesis toward the neutrophil lineage, unveiling the functional role of primed neutrophils within the periodontitis-BM-arthritis axis. Importantly, resolution of local inflammation reverses HSPC adaptations and normalizes neutrophil output. Following ligature removal, neutrophil hyper-responsiveness to LPS and arthritis severity were significantly reduced in comparison to the persistent inflammation group (28dL), although responses remained elevated relative to healthy controls (NL). Given the short lifespan of neutrophils (~2.3 d from BM commitment to release, with a circulatory half-life of 9-18 h), the persistence of primed neutrophils throughout the 14 d recovery period is unlikely 22, 59, 60. Instead, the sustained responsiveness observed in the 14dL/14dR group likely reflects epigenetic or metabolic reprogramming at the progenitor level, consistent with features of trained immunity 15. Collectively, these findings emphasize the importance of early and sustained control of local inflammation to prevent long-term immune dysregulation and distal organ involvement.

This study has several limitations. Although the findings underscore the role of IFN-I in driving neutropoiesis bias, the cellular source of IFN-I and the specific target cell populations mediating this effect remain undefined. Additionally, while bioinformatic analyses suggest that *Rarg* and *Nr2f6* may contribute to IFN-I-mediated neutrophil lineage skewing—potentially through modulation of *Stat1*—further experimental validation is necessary to confirm their functional roles and underlying regulatory mechanisms. Moreover, although the CAIA model was appropriately selected for this study, it does not fully replicate the pathological complexity of human arthritis. Therefore, whether the current findings accurately reflect the clinical interplay between periodontitis and arthritis remains to be determined. Lastly, the potential for periodontitis to exacerbate other inflammatory comorbidities, such as cardiovascular disease and diabetes, via neutropoiesis bias warrants further investigation.

In conclusion, the data presented in this study indicate that periodontitis exacerbates arthritis through IFN-I-mediated neutropoiesis bias, emphasizing the importance of controlling local chronic inflammation in the prevention and management of inflammatory comorbidities.

## Methods

### Mice

C57BL/6 wild type mice were purchased from the Beijing Vital River Laboratory Animal Technology Co., Ltd. (Beijing, China). Ifnar1^-/-^ mice were generated by the Cyagen Bioscience Inc. (Guangzhou, China). B6/SJL (CD45.1) mice were purchased from the Department of Laboratory Animal Science, Peking University Health Science Center (Beijing, China). Eight-week-old male mice were used for all experiments. Mice were housed in specific pathogen-free conditions with a 12:12 h light/dark cycle at a temperature of 24 ± 0.5 °C and a relative humidity of 40-70%. Food and water were provided ad libitum during the experimental period. Animal experiments were approved by the ethics committee of Peking University Health Science Center (approved number: LA2020479).

### Ligature-induced periodontitis

To investigate the impact of experimental periodontitis on BM hematopoiesis, LIP was performed in mice as previously described 61. In brief, bilateral maxillary and mandibular second molars were tied with 5-0 silk ligatures for either 14 d or 28 d to induce periodontitis. Control mice did not undergo ligature placement on their teeth. In some experiments, the ligatures were removed 14 d after placement to enable inflammation resolution. In a subset of these experiments, mice received a secondary challenge of *E. coli* O111:B4 LPS (InVivogen) via intraperitoneal injection at a dose of 1.5 mg/kg body weight to simulate bacteremia. The mice were euthanized 72 h after LPS injection for subsequent analysis.

### Collagen antibody-induced arthritis

Collagen antibody-induced arthritis was induced in mice through intravenous injection of a 5-clone collagen antibody cocktail (Chondrex, Redmond, WA, USA) at a dose of 5 mg. Three days later, mice received an intraperitoneal injection of 50 µg of LPS. Daily clinical scoring was performed visually using a semiquantitative scoring system ranging from 0 (normal) to 4 (maximal inflammation involving multiple joints) per paw 32. The clinical score for each mouse was calculated as the sum of the scores from all four paws, with a maximum score of 16. Hind ankle joint thickness was measured using a thickness gauge. Histological scoring of ankle arthritis severity was conducted according to the previously described evaluation criteria 62. For neutrophil depletion, mice received intraperitoneal injection of 200 µg of rat-anti mouse Ly6G antibody (clone 1A8, Bio X Cell) or isotype control mAbs one day before surgery, followed by subsequent injections every other day at a dose of 100 µg per mouse until 14 d post-surgery.

### Cells preparations and sample collection

For the preparation of BM single-cell suspensions, femurs and tibiae from C57BL/6 mice were flushed with ice-cold RPMI 1640 medium (ThermoFisher). The cells were then passed through a 70 µm nylon mesh sieve to obtain a single-cell suspension for subsequent flow cytometric analysis, mass cytometry, and FACS cell sorting. To collect BM extracellular fluid, femurs and tibiae were flushed with 1 mL of ice-cold PBS, and the supernatant was collected after centrifugation at 500 × g for 5 min at 4 °C. Whole-blood samples were obtained through retrobulbar bleeding. For the preparation of PB single-cell suspensions, whole-blood samples were collected in EDTA anticoagulant tubes, lysed with red blood cell lysis buffer (Solarbio), and filtered through a 70 µm nylon strainer. Serum was obtained by allowing whole-blood samples to stand at room temperature for 30 min without interference, followed by centrifugation at approximately 2000 × g for 15 min, and storage of the samples at -80 °C.

### Flow cytometry and sorting

Flow cytometric analysis was performed by FACS Aria II cytometer (BD Biosciences) and all flow cytometry data were analyzed using FlowJo Software (Tree Star Inc.). For cell surface phenotype analysis, a lineage cocktail (Lin: anti-CD3e (clone 145-2C11), anti-CD11b (clone M1/70), anti-Gr-1 (clone RB6-8C5), anti-B220 (clone RA3-6B2) and anti-TER119 (clone TER-119)), anti-Sca1 (clone D7), anti-cKit (clone 2B8), anti-CD127 (clone A7R34), anti-CD48 (clone HM48-1), anti-CD150 (clone TC15-12F12.2), anti-CD135 (clone A2F10), anti-CD16/32 (clone 93), anti-CD34 (clone RAM34), anti-CD45 (clone 30-F11), anti-CD11b (clone M1/70), anti-Ly6G (clone 1A8), anti-CD3e (clone 145-2C11), anti-B220 (clone RA3-6B2), anti-CD66a (clone MAb-CC1), anti-CD62L (clone MEL-14) were used. Gating strategies for HSPCs were as follows: LSK, Lin^-^Sca-1^+^cKit^+^; LKS^-^, Lin^-^Sca-1^-^cKit^+^; LT-HSC, CD48^-^CD150^+^LSK; ST-HSC, CD48^-^CD150^-^LSK; MPP, CD48^+^CD150^-^LSK; MPP2, Flt3^-^CD48^+^CD150^+^LSK; MPP3, Flt3^-^CD48^+^CD150^-^LSK; MPP4, Flt3^+^CD48^+^CD150^-^LSK; CMP, Lin^-^Sca-1^-^cKit^+^CD16/32^-^CD34^+^; GMP, Lin^-^Sca-1^-^cKit^+^CD16/32^+^CD34^+^; MEP, Lin^-^Sca-1^-^cKit^+^CD16/32^-^CD34^-^; CLP, Lin^-^CD127^+^Sca-1^lo^cKit^lo^. Gating strategies for mature immune cells in PB and BM were as follows: B cells, CD45^+^CD3e^-^B220^+^; neutrophils, CD45^+^CD11b^+^Ly6G^+^. Cell sorting was performed using a FACS Aria Sorp sorter (BD Biosciences). For sorting HSPCs and neutrophils, the following gating strategies were used: HSPCs, Lin^-^cKit^+^; neutrophils, CD45^+^CD11b^+^Ly6G^+^.

### CyTOF sample preparation, acquisition, and analysis

Whole-blood samples were collected using EDTA anticoagulant tubes, gently mixed upside down, labeled, and transported at low temperature to PLTTech Inc. (Hangzhou, China) for CyTOF staining and data acquisition. For each group, whole-blood samples from five mice were pooled into one sample. CyTOF was conducted following previously described protocols 63, 64, and the antibody panel used is listed in Table S2. CyTOF data were analyzed using FlowJo software and R packages. The following sequential filters were applied using FlowJo: removal of normalization beads, selection of singlet cells (Ir191 and Ir193 positive), exclusion of dead cells (high Pt194 signal), and removal of adhesion cells, leaving only live single CD45^+^ immune cells. The .fcs files were imported into R and normalized using arcsinh transformation (cofactor = 5) for downstream analyses. The X-shift clustering algorithm was applied to identify immune cell subsets based on marker expression. To visualize cluster distribution and marker expression, t-SNE dimension reduction was performed. Cell frequency in each cluster was calculated by dividing the number of events in each cluster by the total events in the same sample.

### Bulk RNA-seq and Smart-seq2 library preparation, sequencing, and analysis

Neutrophils were isolated from the BM and PB of mice that underwent 14 d treatment with LIP or Ctrl, using a FACS Aria sorter. Each replicate was prepared by pooling neutrophils from five mice. Total RNA was extracted from the sorted neutrophils using Trizol (Invitrogen), following the manufacturer's instructions. The extracted RNA samples were then sent to LC-Bio Technology (Hangzhou, China) for sequencing and subsequent analysis. For BM neutrophils, bulk RNA-seq libraries were prepared according to the recommended protocol. For PB neutrophils, the Smart-seq2 protocol was employed for sequencing library preparation. The libraries were sequenced on the Illumina NovaSeq™ 6000 platform, generating paired-end 150 bp reads (PE150). Bioinformatics analysis, including quality control, alignment, and differential gene expression analysis, was conducted using standard procedures. Venn plots, four-quadrant plots, heatmaps, and GSEA were performed using the OmicStudio tools available at https://www.omicstudio.cn/tool.

### Cytokine assay

Neutrophils were isolated and sorted from the BM of mice using a FACS Aria Sorp sorter (BD Biosciences). The sorted neutrophils were then maintained in RPMI 1640 medium (ThermoFisher) supplemented with 10% fetal bovine serum (FBS) for 30 min. Subsequently, the neutrophils were seeded into 12-well plates (1 × 10^6^ cells/well) and stimulated with 150 ng/mL *E. coli* O111:B4 LPS (InVivogen) for 17 h. The supernatant was collected for measurement of IL-1β, IL-6, and TNF-α concentrations using a mouse ELISA kit (absin), following the manufacturer's instructions. The Mouse IFN-beta Quantikine ELISA Kit (R&D Systems) and Mouse IFN-alpha All Subtype Quantikine ELISA Kit (R&D Systems) were utilized to determine the concentrations of IFNα and IFNβ in serum and BM extracellular fluid, respectively, following the manufacturer's instructions. For the Luminex assay, the neutrophil supernatant, serum, and BM extracellular fluid were collected as mentioned above. The assay was conducted at Wayen Biotechnologies Shanghai, Inc., following the manufacturer's protocol, using the Bio-Plex Pro Mouse Cytokine 23-plex Assay (#M60009RDPD) with the Luminex 200 system (Austin, TX, USA).

### IFN-I bioassay

The ISRE luciferase reporter plasmid was constructed by incorporating the ISRE sequence into the Lenti-luciferase vector using a seamless cloning kit (Sangon Biotech) with Gibson technology. The resulting reporter plasmid was co-transfected with pSPAX2 and pMD2.G plasmids into HEK 293T cells at a ratio of 5:3:2. Lentivirus present in the supernatant was collected 3 d post-transfection and added to HEK 293T cells. After an additional 2 d, stably transfected cells were selected using hygromycin (100 μg/mL). To determine the levels of mouse IFN-I, HEK 293T-ISRE-Luc cells were incubated with serum or BM fluid for 24 h. Subsequently, the cell lysates were harvested and luciferase activity was measured using a luciferase assay system (Promega) and an EnSpire & dispenser (PerkinElmer), following the manu-facturer's instructions.

### Neutrophil ROS and NETs assay

After a 17 h stimulation with LPS, we assessed the ROS levels of neutrophils. To measure ROS levels, the medium was aspirated, and the cells were resuspended in PBS containing 5 µM H2DCFDA (MedChemExpress) and incubated for 30 min at 37 °C, protected from light. Subsequently, the cells were carefully washed twice with PBS, resuspended in fresh medium, and immediately analyzed using a flow cytometer. For the NETs assay, neutrophils were seeded in confocal dishes (1 × 10^6^ cells per dish) and allowed to adhere for 30 min at 37 °C. The cells were then stimulated with 10 μg/mL *E. coli* O111:B4 LPS (InVivogen) for 3 h at 37 °C to induce NET formation. The samples were stained and analyzed according to a previously described protocol 65. Primary antibodies anti-citrullinated histone H3 (ab5103, Abcam; 1:500) and anti-MPO (AF3667, R&D systems; 1:40) were used. Following PBS washes, the samples were incubated with anti-rabbit Alexa Fluor 647-conjugated antibody (ab150075, Abcam; 1:500), anti-goat FITC-conjugated antibody (ab6881, Abcam; 1:1000), and DAPI (Invitrogen; 1:1000) for 1 h in the dark at room temperature. Images were acquired using a TCS-SP8 STED 3X microscope and analyzed with Fiji (v 2.0.0). The percentage of CitH3 signal was normalized to the MPO signal, and the %CitH3/MPO values obtained from different regions were averaged and plotted.

### Histological and immunostaining assays

Mouse hind paws and femur were fixed with 4% paraformaldehyde, decalcified in EDTA solution, dehydrated in a graded series of alcohol, paraffin embedded and tissue sectioned to 5 µm for histological evaluation. For hind paw, deparaffinized, and stained with HE, safranin-O/fast green and toluidine blue for scoring histopathology of inflammatory arthritis. For immunofluorescence of hind paws, slides were deparaffinized and subjected to antigen retrieval, then permeabilized, blocked, incubated antibodies, and stained nuclei, as described in the NETs assay above. For multicolor immunohistochemistry (mIHC) of femurs, tissue slides were deparaffinized and treated by microwave to induce antigen retrieval using citric acid solution for 15 min. Primary antibodies, including mouse hematopoietic lineage antibody cocktail (88-7772-72, invitrogen, 1:200), anti-CD117 (14-1171-82, invitrogen, 1:1000), anti-Ly-6A/E (14-5981-82, invitrogen, 1:500), anti-CD16/32 (14-0161-82, invitrogen, 1:500) and anti-CD34 (14-0341-82, invitrogen, 1:1000), were used. Subsequently, the slides were incubated with secondary antibodies (HRP-anti-rat IgG, ZSGB-Bio, PV-9004) at room temperature for 20 min. Heat-induced epitope retrieval was performed after each staining cycle to remove all antibodies, including primary and secondary antibodies. Multiplex immunofluorescence staining was performed using the AlphaTSA Multiplex IHC Kit (AXT36100031, AlphaX). Nuclei were counterstained with DAPI for 10 min, and the samples were mounted in mounting medium. Multispectral images were scanned with ZEISS AXIOSCAN 7, and quantification of cells of interest was performed using Halo software (v.3.4; Indica Labs).

### Micro-CT analysis

The hind paws of mice were fixed in 4% paraformaldehyde for 24 h and subjected to scanning using a high-resolution Inveon microtomography system (Siemens, Munich, Germany). BMD analysis was performed, and 3D reconstruction was conducted based on the processed images using Scanco software.

### Neutrophil chemotaxis assays

Neutrophil chemotaxis was evaluated using Transwell 24-well plates (Corning) with 5 μm porous membranes. For the chemotaxis assay, neutrophils were seeded in the upper chamber (1 × 10^4^ cells/well), while RPMI 1640 medium containing 5 μM fMLP (MedChemExpress) and 10% FBS was added to the lower chamber. Following a 6 h incubation, unmigrated cells on the upper surface of the membrane were gently removed using a rubber scraper. Migrated cells on the lower surface of the membrane were fixed with 4% paraformaldehyde, stained with crystal violet, and counted in five random fields using an optical microscope (200 ×).

### Neutrophil tracing

To evaluate the migratory capacity of neutrophils derived from Ctrl and LIP mice towards the inflamed ankle joint in the CAIA model, we employed a previously described method 66. Following the isolation of neutrophils, direct cell membrane labeling was performed using CellVue NIR815 dye (LI-COR) according to the manufacturer's instructions. Subsequently, the neutrophils were intravenously injected into each mouse at a volume of 100 µL per injection. Under pentobarbital anesthesia, mice were subjected to fluorescent imaging using excitation and emission filters of 780/845 nm. Epi-fluorescent images were acquired using an IVIS Lumina III (PerkinElmer) at 20 h post cell administration. To optimize signal sensitivity, all images were acquired under the same field of view using autoexposure settings, with an f/Stop of 2 and pixel binning of 8.

### Adoptive transfer of neutrophils

BM neutrophils from donor mice, treated with Ctrl or LIP, were isolated and sorted. Each recipient mouse, during the CAIA modeling phase (on day 0 and day 4), received two tail vein injections of 5 × 10^6^ neutrophils. To assess the impact of IFN-I signaling blockage on arthritis exacerbation by periodontitis-activated neutrophils, CAIA mice were injected intraperitoneally with either anti-IFNα/β or isotype control (500 µg/dose) 1 h after receiving LIP-primed neutrophils. BM neutrophils, harvested from Ctrl or LIP-treated donor mice (CD45.2) after 14 d, were injected into CAIA mice (CD45.1). Synovial tissues from the knee and ankle joints were collected 20 h later to analyze neutrophil infiltration.

### CFU-assays

CFU assays were performed using MethoCult™ GF M3434 medium (STEMCELL Technologies) in 35-mm culture dishes. Briefly, 3 × 10^4^ freshly isolated BM cells were uniformly suspended in 3 mL of MethoCult™ medium per dish. To assess the effects of inflammatory microenvironments, BM cells were resuspended in 100 μL of serum or BM extracellular fluid collected from LIP or Ctrl mice prior to mixing with MethoCult™ medium. In parallel, cells were resuspended in 100 μL of either complete medium without IFN-I or complete medium supplemented with 30 pg/mL recombinant IFN-I, and then mixed with 3 mL of MethoCult™. All conditions were plated in duplicate. After 7 d of incubation at 37 °C in a humidified 5% CO₂ atmosphere, colonies were examined under an inverted microscope and classified based on morphological criteria, following the manufacturer's instructions.

### RT-qPCR

At 14 d post-ligation, mice were euthanized and gingival tissues were harvested. In addition, BM-derived LSK cells, CMPs, and GMPs were sorted by flow cytometry. Total RNA was extracted from tissues and sorted cells using TRIzol reagent (Invitrogen, USA) and reverse-transcribed into cDNA using Evo M-MLV RT Premix (AG11706, Accurate Biotech-nology), following the manufacturer's instructions. RT-qPCR was performed using Hieff UNICON® Universal Blue qPCR SYBR Green Master Mix (11184ES08, Yeasen, China) on a QuantStudio system (Thermo Fisher). Relative gene expression was normalized to *Gapdh* mRNA levels using the 2^-ΔΔCt^ method. Primer sequences used for amplification are listed in Table S3.

### Antibody-mediated inhibition of IFN-I signaling

To inhibit IFN-I signaling, mice were intraperitoneally injected with a neutralizing anti-mouse antibody against the receptor for IFNα/β (clone MAR1-5A3, Biolegend) or an isotype control (clone MOPC-21, Biolegend) one day before and on the same day as silk ligation or sham surgery (500 µg/dose) 37. After 14 d from the latter injection, the immune cell composition of BM and PB was analyzed, the secondary stress capacity of neutrophils was assessed, and the effect of IFN-I signaling on arthritis with periodontitis was evaluated by inducing an arthritis model.

### Chromium Next Single cell multiome ATAC + gene expression

Lin^-^ckit^+^ cells from the BM of mice, which underwent 14 d silk ligation or remained untreated for 14 d, were sorted using a FACS Aria Sorp sorter (BD Biosciences). A single-cell nuclear suspension was prepared from the sorted cells, and cell counting and viability assessment were performed using Countstar. Cells with a viability of 0%-5% were considered for further analysis, and the cell concentration was adjusted to the desired concentration of 2 × 10^3^ cells/μL. Isolated nuclei from Lin^-^ckit^+^ cells were transposed and loaded onto the Chromium Next GEM Chip J (10X Genomics). Subsequently, the cell chip was loaded onto a Chrome Controller, and the library was sequenced on a NovaSeq 6000 platform (Illumina) following the manufacturer's instructions. The sequencing was performed by CapitalBio Technology in Beijing.

### Single-cell multiomics data processing

The raw multiomic data was input into the CellRanger-ARC-2.0.0 pipeline to perform sequence alignment and generate single-cell feature counts. Reads were aligned to the GRCm38 (mm10) reference genome. Subsequently, quality control and preprocessing steps were conducted using Seurat (v.4.0.3) and Signac (v.1.3.0). For scRNA-seq data, cells with a low number of features (< 200) or a high fraction of mitochondrial genes (> 25%) were filtered out. For scATAC-seq data, cells with low fragment counts (< 1 000) or high fragment counts (> 50 000) were excluded. Finally, the remaining cells obtained after the integration of scATAC and scRNA data were selected for downstream analysis.

### Dimensionality reduction and clustering of all the cells

For scRNA analysis, dimensionality reduction was performed using principal component analysis (PCA), and the top 30 principal components were utilized to generate clusters. For scATAC analysis, dimensionality reduction was accomplished through latent semantic indexing (LSI), employing parameter settings at dims = 2:30. Subsequently, a WNN analysis was employed to integrate the dimensionality reduction results from PCA and LSI, facilitating the clustering of all single cells 67. Based on the WNN analysis, cells were further grouped into 16 distinct clusters using the 'FindClusters' function with a resolution of 0.8. To explore the cellular heterogeneity of GMP in more detail, we applied the 'FindClusters' function with a resolution of 0.6 to group GMP into distinct clusters.

### Identifying differential gene expression and gene scores across cell types

Marker genes for each cluster of HSPCs were identified using the 'FindAllMarkers' function in Seurat. Predicted gene scores were determined based on peak accessibility. Due to space limitations, we only reported the representative markers for each cell type.

### Cell cycle phase determination

The cell cycle phase of individual cells was determined using the 'cyclone' algorithm. Each cell received a phase-specific score (G2/M, S, and G1) calculated using raw count data prior to gene filtering, enabling accurate phase assignment. To analyze the cell cycle, cells were fixed, permeabilized with Foxp3/TF Buffer Set (eBioscience), and stained with anti-Ki-67 (clone 16A8, Biolegend). After washing, cells were stained with DAPI (Invitrogen) and analyzed by flow cytometry.

### GO enrichment analysis of single-cell RNA-seq

To define marker genes for each cluster and identify differential genes between the two groups, we employed the 'FindAllMarkers' function in Seurat (LogFC > 0.25, min.pct > 0.25). Subsequently, we performed GO enrichment analysis and visualization on these genes using clusterProfiler 68.

### Inference of pseudotime and trajectory

Cell differentiation trajectories for representative HSPC subsets (LSK, CMP, GMP, NeuP, CLP, and pro_B), as well as all GMP subsets, were constructed based on gene expression profiles using Monocle2 69. Pseudotime was divided into 50 bins for the stacked plot of HSPC subclusters 70. Stacked plots were computed and plotted along pseudotime at each sample for two HSPC fates: lymphoid (Monocle states 1, 3, 4, and 6) and myeloid (Monocle states 1, 3, 4, and 5). Additionally, lymphoid and myeloid trajectories were created using the 'additional trajectory' function, and pseudotime values were plotted on a UMAP embedding using the 'plotTrajectory' function.

### Linking gene regulatory elements and gene expression in the pseudotime trajectory

To enable aggregate analysis along pseudotime, we sorted cells based on their pseudotime scores and grouped 50 adjacent cells into pseudobulk samples. We linked peaks to cluster-specific genes by applying a correlation-based approach to these pseudobulk samples aggregating scATAC and scRNA counts. Utilizing the 'FindAllMarkers' function, we identified cluster-specific genes from the scRNA data of cells included in the pseudotime trajectory. We established high-confidence peak-to-gene links specific to the pseudotime trajectory by associating peaks located 1-250 kb from the TSS of protein coding and lincRNA genes to the respective genes. Pearson correlation coefficients and corresponding *P*-values were calculated for each peak-gene pair based on CPM-normalized accessibility and gene expression data. We considered pairs with |PCC| ≥ 0.3 and *P* < 0.05 as high-confidence links. These links were then clustered using k-means clustering (k = 6) based on z-score-scaled expression levels of the associated genes. GSEA was performed for the genes in these clusters using the 'clusterprofiler' R package, and TF motif enrichment analysis was conducted for the peaks in these clusters using the 'motifmatchr' R package 71.

### Matching TF motif accessibility to TF gene scores in the pseudotime trajectory

Across the same pseudotime-pseudobulk samples that were used for peak-gene linking, we computed the pairwise pearson correlation coefficients between TF motif accessibility and TF gene scores for each pseudobulk sample. To identify positive TF regulators, we conducted a matching analysis by associating gene scores with motif accessibility across the pseudotime trajectory 71.

### Calculation of motif synergy and correlation scores

We calculated the pairwise synergy scores and correlation coefficients between motif clusters using the 'getAnnotationSynergy' and 'getAnnotation-Correlation' chromVAR function 72, respectively.

### TF footprinting and tracks plotting

We conducted TF motif footprinting to precisely predict the binding site of a TF at specific genomic loci. This analysis was implemented using the 'Footprint' function in Signac, utilizing the TF motif information sourced from the JASPAR database 73. Additionally, we plotted the genome browser tracks of peaks using the 'CoveragePlot' function in Signac 74.

### TF-gene interaction network analysis

We identified the TSS positions for each gene within the IFN-I signaling pathway gene set (GO:0060337) using the mouse .gtf file. Subsequently, we selected a genomic region spanning 2 000 base pairs upstream and 500 base pairs downstream of the TSS for each gene to encompass potential regulatory elements. Computational predictions were then employed to evaluate the binding and regulatory potential of the TFs *Rarg* and *Nr2f6* on these DNA sequences, thereby generating a list of target genes. Next, we performed protein-protein interaction analysis using the stringDB database 75 to explore the interactions between the identified TFs and the target genes. The resulting interactions were visualized using Cytoscape software 76.

### Statistics

Animals were randomly allocated to either the vehicle or treatment groups. Blinding procedures were employed to evaluate the severity of paw inflammation and the histopathology of arthritis. Apart from the omics sequencing data, all experiments were performed in duplicate. A two-tailed Student's *t*-test was used to compare data between two independent groups. For comparisons involving three or more groups with a single variable, one-way ANOVA was performed, followed by Tukey's post hoc test for multiple comparisons. Statistical analysis was conducted using GraphPad Prism 8.0.2 software. All data were presented as mean ± SD, and statistical significance was defined as *P* < 0.05.

## Abbreviations

HSC: hematopoietic stem cell
LT-HSC: long-term hematopoietic stem cell
ST-HSC: short-term hematopoietic stem cell
MPP: multipotent progenitor cell
CMP: common myeloid progenitor
GMP: granulocyte-monocyte progenitor
CLP: common lymphoid progenitor
pro_B: progenitor B cells
pro_NK/T: progenitor NK/T cells
NeuP: neutrophil progenitors
cMoP: monocyte progenitors
cDCP: conventional dendritic cell progenitors
pDCP: plasmacytoid dendritic cell progenitors
MEP: megakaryocyte-erythrocyte progenitors
EryP: erythrocyte progenitors
MkP: megakaryocyte progenitors
BaP: basophil progenitors
CFU-G: colony-forming unit-granulocyte
CFU-M: colony-forming unit-monocyte
CFU-GM: colony-forming unit-granulocyte and monocyte
CFU-GEMM: colony-forming unit-granulocyte, erythrocyte, monocyte, and megakaryocyte

## References

[1] Agirman G, Yu KB, Hsiao EY (2021). Signaling inflammation across the gut-brain axis. Science.

[2] Furman D, Campisi J, Verdin E, Carrera-Bastos P, Targ S, Franceschi C (2019). Chronic inflammation in the etiology of disease across the life span. Nat Med.

[3] Marcelin G, Gautier EL, Clement K (2022). Adipose Tissue Fibrosis in Obesity: Etiology and Challenges. Annu Rev Physiol.

[4] Hemminki K, Sundquist K, Sundquist J, Forsti A, Liska V, Hemminki A (2023). Personal comorbidities and their subsequent risks for liver, gallbladder and bile duct cancers. Int J Cancer.

[5] Decramer M, Janssens W (2013). Chronic obstructive pulmonary disease and comorbidities. Lancet Respir Med.

[6] Genco RJ, Sanz M (2020). Clinical and public health implications of periodontal and systemic diseases: An overview. Periodontol 2000.

[7] Hajishengallis G (2015). Periodontitis: from microbial immune subversion to systemic inflammation. Nat Rev Immunol.

[8] Konkel JE, O'Boyle C, Krishnan S (2019). Distal Consequences of Oral Inflammation. Front Immunol.

[9] Potempa J, Mydel P, Koziel J (2017). The case for periodontitis in the pathogenesis of rheumatoid arthritis. Nat Rev Rheumatol.

[10] Schenkein HA, Papapanou PN, Genco R, Sanz M (2020). Mechanisms underlying the association between periodontitis and atherosclerotic disease. Periodontol 2000.

[11] Peres MA, Macpherson LMD, Weyant RJ, Daly B, Venturelli R, Mathur MR (2019). Oral diseases: a global public health challenge. Lancet.

[12] Skou ST, Mair FS, Fortin M, Guthrie B, Nunes BP, Miranda JJ (2022). Multimorbidity. Nat Rev Dis Primers.

[13] Vrinzen CEJ, Delfgou L, Stadhouders N, Hermens R, Merkx MAW, Bloemendal HJ (2023). A Systematic Review and Multilevel Regression Analysis Reveals the Comorbidity Prevalence in Cancer. Cancer Res.

[14] Dewan P, Ferreira JP, Butt JH, Petrie MC, Abraham WT, Desai AS (2023). Impact of multimorbidity on mortality in heart failure with reduced ejection fraction: which comorbidities matter most? An analysis of PARADIGM-HF and ATMOSPHERE. Eur J Heart Fail.

[15] Li XF, Wang H, Yu X, Saha G, Kalafati L, Ioannidis C (2022). Maladaptive innate immune training of myelopoiesis links inflammatory comorbidities. Cell.

[16] Chavakis T, Mitroulis I, Hajishengallis G (2019). Hematopoietic progenitor cells as integrative hubs for adaptation to and fine-tuning of inflammation. Nat Immunol.

[17] Gaffen SL, Moutsopoulos NM (2020). Regulation of host-microbe interactions at oral mucosal barriers by type 17 immunity. Sci Immunol.

[18] Collins A, Mitchell CA, Passegué E (2021). Inflammatory signaling regulates hematopoietic stem and progenitor cell development and homeostasis. J Exp Med.

[19] Pietras EM (2017). Inflammation: a key regulator of hematopoietic stem cell fate in health and disease. Blood.

[20] Manz MG, Boettcher S (2014). Emergency granulopoiesis. Nat Rev Immunol.

[21] Schultze JL, Mass E, Schlitzer A (2019). Emerging Principles in Myelopoiesis at Homeostasis and during Infection and Inflammation. Immunity.

[22] Overbeeke C, Tak T, Koenderman L (2022). The journey of neutropoiesis: how complex landscapes in bone marrow guide continuous neutrophil lineage determination. Blood.

[23] Ling MR, Chapple ILC, Matthews JB (2016). Neutrophil superoxide release and plasma C-reactive protein levels pre- and post-periodontal therapy. J Clin Periodontol.

[24] Wong SL, Demers M, Martinod K, Gallant M, Wang Y, Goldfine AB (2015). Diabetes primes neutrophils to undergo NETosis, which impairs wound healing. Nat Med.

[25] Silvestre-Roig C, Fridlender ZG, Glogauer M, Scapini P (2019). Neutrophil Diversity in Health and Disease. Trends Immunol.

[26] Chen S, Lake BB, Zhang K (2019). High-throughput sequencing of the transcriptome and chromatin accessibility in the same cell. Nat Biotechnol.

[27] Ma S, Zhang B, LaFave LM, Earl AS, Chiang Z, Hu Y (2020). Chromatin Potential Identified by Shared Single-Cell Profiling of RNA and Chromatin. Cell.

[28] Ranzoni AM, Tangherloni A, Berest I, Riva SG, Myers B, Strzelecka PM (2021). Integrative Single-Cell RNA-Seq and ATAC-Seq Analysis of Human Developmental Hematopoiesis. Cell Stem Cell.

[29] Laurenti E, Gottgens B (2018). From haematopoietic stem cells to complex differentiation landscapes. Nature.

[30] Velten L, Haas SF, Raffel S, Blaszkiewicz S, Islam S, Hennig BP (2017). Human haematopoietic stem cell lineage commitment is a continuous process. Nat Cell Biol.

[31] Koziel J, Potempa J (2022). Pros and cons of causative association between periodontitis and rheumatoid arthritis. Periodontol 2000.

[32] Khachigian LM (2006). Collagen antibody-induced arthritis. Nat Protoc.

[33] Apel F, Zychlinsky A, Kenny EF (2018). The role of neutrophil extracellular traps in rheumatic diseases. Nat Rev Rheumatol.

[34] Wigerblad G, Kaplan MJ (2023). Neutrophil extracellular traps in systemic autoimmune and autoinflammatory diseases. Nat Rev Immunol.

[35] King KY, Goodell MA (2011). Inflammatory modulation of HSCs: viewing the HSC as a foundation for the immune response. Nat Rev Immunol.

[36] Christ A, Günther P, Lauterbach MAR, Duewell P, Biswas D, Pelka K (2018). Western Diet Triggers NLRP3-Dependent Innate Immune Reprogramming. Cell.

[37] Kalafati L, Kourtzelis I, Schulte-Schrepping J, Li XF, Hatzioannou A, Grinenko T (2020). Innate Immune Training of Granulopoiesis Promotes Anti-tumor Activity. Cell.

[38] Yvan-Charvet L, Ng LG (2019). Granulopoiesis and Neutrophil Homeostasis: A Metabolic, Daily Balancing Act. Trends Immunol.

[39] Zhang DX, Glass CK (2013). Towards an understanding of cell-specific functions of signal-dependent transcription factors. J Mol Endocrinol.

[40] Evrard M, Kwok IWH, Chong SZ, Teng KWW, Becht E, Chen JM (2018). Developmental Analysis of Bone Marrow Neutrophils Reveals Populations Specialized in Expansion, Trafficking, and Effector Functions. Immunity.

[41] Ng LG, Ostuni R, Hidalgo A (2019). Heterogeneity of neutrophils. Nat Rev Immunol.

[42] Cowland JB, Borregaard N (2016). Granulopoiesis and granules of human neutrophils. Immunol Rev.

[43] Paul F, Arkin Y, Giladi A, Jaitin DA, Kenigsberg E, Keren-Shaul H (2015). Transcriptional Heterogeneity and Lineage Commitment in Myeloid Progenitors. Cell.

[44] Giladi A, Paul F, Herzog Y, Lubling Y, Weiner A, Yofe I (2018). Single-cell characterization of haematopoietic progenitors and their trajectories in homeostasis and perturbed haematopoiesis. Nat Cell Biol.

[45] Duarte PM, da Rocha M, Sampaio E, Mestnik MJ, Feres M, Figueiredo LC (2010). Serum Levels of Cytokines in Subjects with Generalized Chronic and Aggressive Periodontitis Before and After Non-Surgical Periodontal Therapy: A Pilot Study. J Periodontol.

[46] Andrukhov O, Ulm C, Reischl H, Nguyen PQ, Matejka M, Rausch-Fan X (2011). Serum Cytokine Levels in Periodontitis Patients in Relation to the Bacterial Load. J Periodontol.

[47] Wright HJ, Matthews JB, Chapple ILC, Ling-Mountford N, Cooper PR (2008). Periodontitis associates with a type 1 IFN signature in peripheral blood neutrophils. J Immunol.

[48] Kim YM, Kim H, Lee S, Kim S, Lee JU, Choi Y (2020). Airway G-CSF identifies neutrophilic inflammation and contributes to asthma progression. Eur Respir J.

[49] Reynaud D, Pietras E, Barry-Holson K, Mir A, Binnewies M, Jeanne M (2011). IL-6 Controls Leukemic Multipotent Progenitor Cell Fate and Contributes to Chronic Myelogenous Leukemia Development. Cancer Cell.

[50] Lv M, Chen M, Zhang R, Zhang W, Wang C, Zhang Y (2020). Manganese is critical for antitumor immune responses via cGAS-STING and improves the efficacy of clinical immunotherapy. Cell Res.

[51] Xian H, Watari K, Sanchez-Lopez E, Offenberger J, Onyuru J, Sampath H (2022). Oxidized DNA fragments exit mitochondria via mPTP- and VDAC-dependent channels to activate NLRP3 inflammasome and interferon signaling. Immunity.

[52] Chen K, Liu J, Cao X (2017). Regulation of type I interferon signaling in immunity and inflammation: A comprehensive review. J Autoimmun.

[53] Loos BG, Craandijk J, Hoek FJ, Wertheim-van Dillen PM, van der Velden U (2000). Elevation of systemic markers related to cardiovascular diseases in the peripheral blood of periodontitis patients. J Periodontol.

[54] Gaudilliere DK, Culos A, Debali K, Tsai AS, Ganio EA, Choi WM (2019). Systemic Immunologic Consequences of Chronic Periodontitis. J Dent Res.

[55] Irwandi RA, Kuswandani SO, Harden S, Marletta D, D'Aiuto F (2022). Circulating inflammatory cell profiling and periodontitis: A systematic review and meta-analysis. J Leukoc Biol.

[56] Jorch SK, Kubes P (2017). An emerging role for neutrophil extracellular traps in noninfectious disease. Nat Med.

[57] Herrero-Cervera A, Soehnlein O, Kenne E (2022). Neutrophils in chronic inflammatory diseases. Cell Mol Immunol.

[58] Silvestre-Roig C, Braster Q, Ortega-Gomez A, Soehnlein O (2020). Neutrophils as regulators of cardiovascular inflammation. Nat Rev Cardiol.

[59] Hidalgo A, Chilvers ER, Summers C, Koenderman L (2019). The Neutrophil Life Cycle. Trends Immunol.

[60] Pillay J, den Braber I, Vrisekoop N, Kwast LM, de Boer RJ, Borghans JAM (2010). *In vivo* labeling with 2H2O reveals a human neutrophil lifespan of 5.4 days. Blood.

[61] Kitamoto S, Nagao-Kitamoto H, Jiao Y, Gillilland MG 3rd, Hayashi A, Imai J (2020). The Intermucosal Connection between the Mouth and Gut in Commensal Pathobiont-Driven Colitis. Cell.

[62] Hayer S, Vervoordeldonk MJ, Denis MC, Armaka M, Hoffmann M, Backlund J (2021). 'SMASH' recommendations for standardised microscopic arthritis scoring of histological sections from inflammatory arthritis animal models. Ann Rheum Dis.

[63] Mei HE, Leipold MD, Maecker HT (2016). Platinum-conjugated antibodies for application in mass cytometry. Cytometry A.

[64] Mei HE, Leipold MD, Schulz AR, Chester C, Maecker HT (2015). Barcoding of live human peripheral blood mononuclear cells for multiplexed mass cytometry. J Immunol.

[65] Moreira-Teixeira L, Stimpson PJ, Stavropoulos E, Hadebe S, Chakravarty P, Ioannou M (2020). Type I IFN exacerbates disease in tuberculosis-susceptible mice by inducing neutrophil-mediated lung inflammation and NETosis. Nat Commun.

[66] Leslie J, Robinson SM, Oakley F, Luli S (2021). Non-invasive synchronous monitoring of neutrophil migration using whole body near-infrared fluorescence-based imaging. Sci Rep.

[67] Hao Y, Hao S, Andersen-Nissen E, Mauck WM 3rd, Zheng S, Butler A (2021). Integrated analysis of multimodal single-cell data. Cell.

[68] Yu G, Wang LG, Han Y, He QY (2012). clusterProfiler: an R package for comparing biological themes among gene clusters. OMICS.

[69] Qiu X, Mao Q, Tang Y, Wang L, Chawla R, Pliner HA (2017). Reversed graph embedding resolves complex single-cell trajectories. Nat Methods.

[70] Herault L, Poplineau M, Mazuel A, Platet N, Remy E, Duprez E (2021). Single-cell RNA-seq reveals a concomitant delay in differentiation and cell cycle of aged hematopoietic stem cells. BMC Biol.

[71] Trevino AE, Muller F, Andersen J, Sundaram L, Kathiria A, Shcherbina A (2021). Chromatin and gene-regulatory dynamics of the developing human cerebral cortex at single-cell resolution. Cell.

[72] Schep AN, Wu B, Buenrostro JD, Greenleaf WJ (2017). chromVAR: inferring transcription-factor-associated accessibility from single-cell epigenomic data. Nat Methods.

[73] Castro-Mondragon JA, Riudavets-Puig R, Rauluseviciute I, Lemma RB, Turchi L, Blanc-Mathieu R (2022). JASPAR 2022: the 9th release of the open-access database of transcription factor binding profiles. Nucleic Acids Res.

[74] Stuart T, Srivastava A, Madad S, Lareau CA, Satija R (2021). Single-cell chromatin state analysis with Signac. Nat Methods.

[75] Szklarczyk D, Gable AL, Nastou KC, Lyon D, Kirsch R, Pyysalo S (2021). The STRING database in 2021: customizable protein-protein networks, and functional characterization of user-uploaded gene/measurement sets. Nucleic Acids Res.

[76] Doncheva NT, Morris JH, Gorodkin J, Jensen LJ (2019). Cytoscape StringApp: Network Analysis and Visualization of Proteomics Data. J Proteome Res.

