# Supplementary material for: IFN-I-mediated neutropoiesis bias drives neutrophil priming and inflammatory comorbidities
Source: Theranostics. 2025 May 6;15(13):6058–81. doi: 10.7150/thno.110859 (PMC12159751; doi:10.7150/thno.110859)
Supplement: Supplementary file 1 — Supplementary figures and tables. [file thnov15p6058s1.pdf]

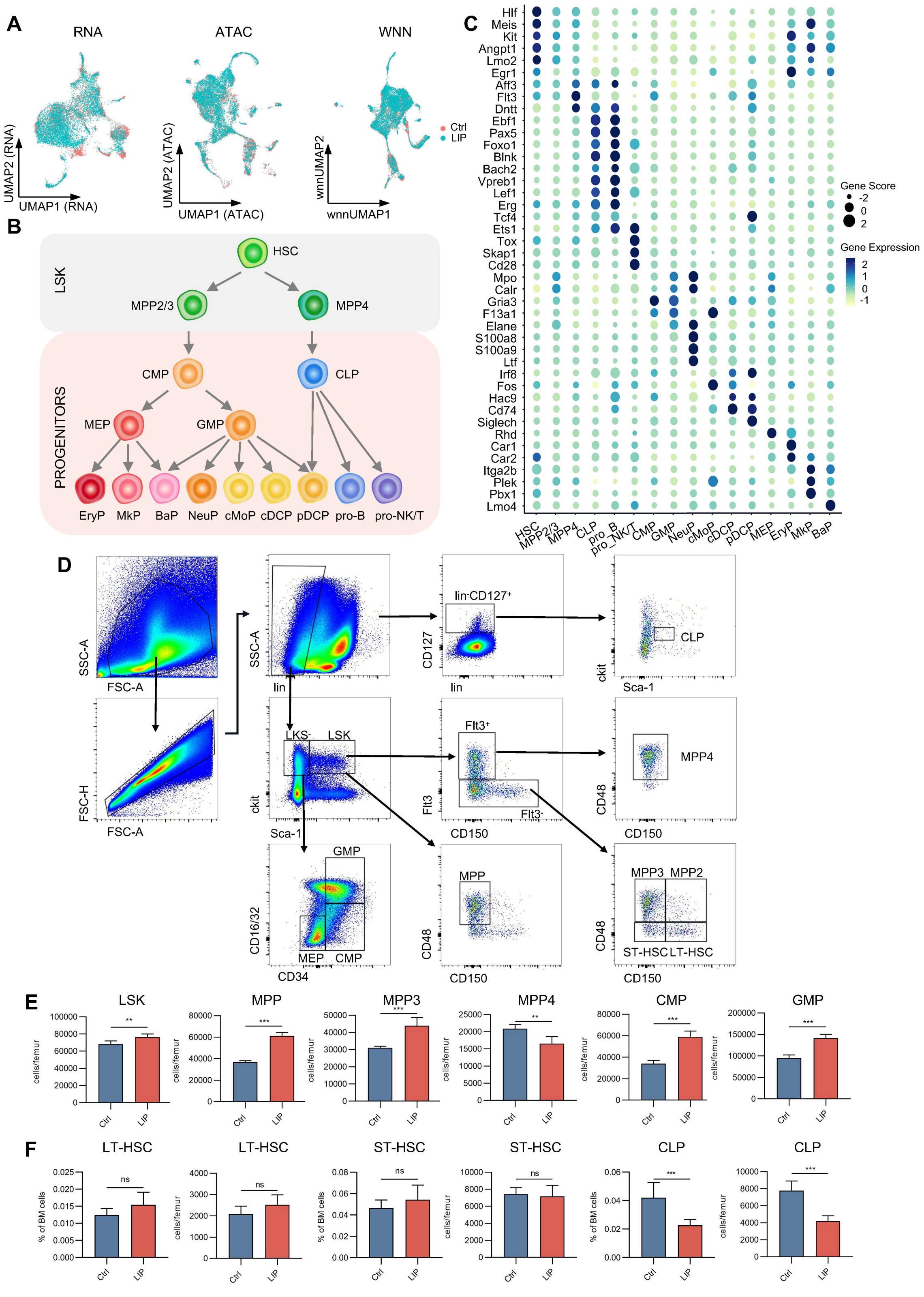

Figure S1. Comprehensive analysis of HSPCs, related to Figure 1. (A) UMAP visualizations for Ctrl vs LIP were computed using RNA, ATAC, and WNN analysis,

respectively. **(B)** Schematic diagram of the hematopoietic-lineage differentiation stages included in this study. **(C)** Dot plot of gene enrichment within clusters; colors represent the mean gene expression, and dot size represents the mean gene score. **(D)** Gating strategy for HSPCs. Cells were first gated based on forward scatter (FSC-A) and side scatter (SSC-A) to exclude debris. Singlets were gated using FSC-H against FSC-A, and lineage-committed cells were excluded. Within the lineage-negative population, cells were gated as LSK defined as double positive for cKit and Sca-1. Within the LSK population, cells were further divided into $Flt3^+$ and $Flt3^-$ population based on CD150 and Flt3 expression. MPP4 was defined as $Flt3^+CD150^-CD48^+$. The $Flt3^-$ population was divided into MPP2, MPP3, ST-HSC, and LT-HSC by their surface expression of CD150 and CD48. In addition, cells were gated as MPP defined as $CD150^-CD48^+$ in the LSK population. In a second strategy, $c\text{-}Kit^+Sca\text{-}1^-$ cells were defined as the LKS^- population and further gated based on CD34 and CD16/32 to define CMP, GMP, and MEP. Finally, lineage-negative cells were gated based on CD127. Lin^-CD127^+ cells were further denoted as CLP based on $Sca\text{-}1^{lo}$ and $c\text{-}Kit^{lo}$ expression. **(E)** Absolute cell numbers of LSK, MPP, MPP3, MPP4, CMP and GMP in total BM cells. **(F)** Frequencies and absolute cell numbers of LT-HSC, ST-HSC and CLP in total BM cells. Data are presented as the mean ± SD from at least three independent experiments; n = 6 mice/group (E-F). *P* values were calculated using two-tailed Student's *t* test; **P* < 0.05, ***P* < 0.01, ****P* < 0.001, ns indicates no significant difference.

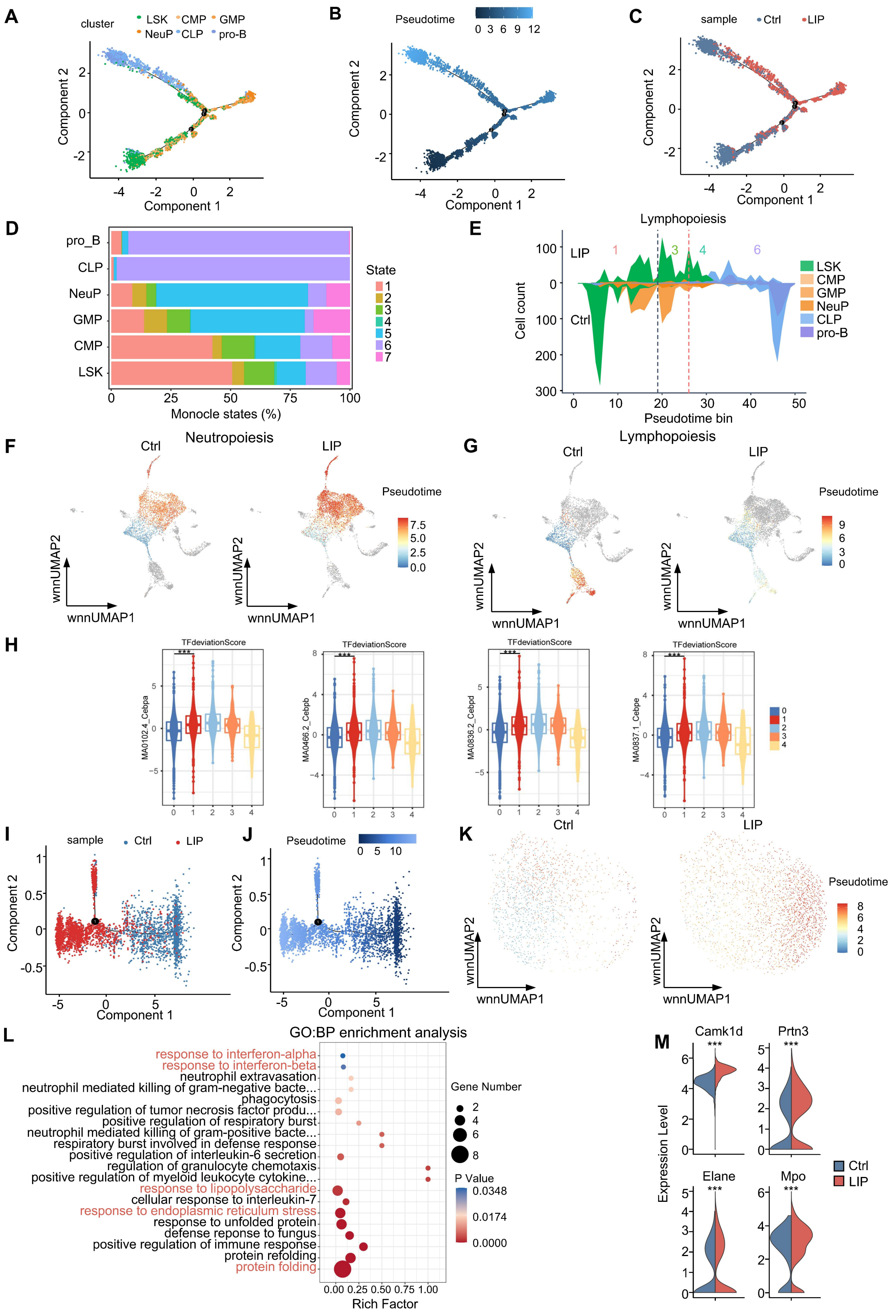

A
cluster
LSK CMP GMP NeuP CLP pro-B
Component 2
Component 1
B
Pseudotime
0 3 6 9 12
C
sample
Ctrl
LIP
D
pro_B
CLP
NeuP
GMP
CMP
LSK
Monocle states (%)
State
E
Lymphopoiesis
LIP
Ctrl
Cell count
Pseudotime bin
F
Neutropoiesis
Ctrl
LIP
Pseudotime
wnnUMAP1
wnnUMAP2
G
Lymphopoiesis
H
TFdeviationScore
MA0102.4_Cebpa
MA0466.2_Cebpb
MA0836.2_Cebpd
MA0837.1_Cebpe
I
J
K
L
GO:BP enrichment analysis
response to interferon-alpha
response to interferon-beta
neutrophil extravasation
neutrophil mediated killing of gram-negative bacte...
phagocytosis
positive regulation of tumor necrosis factor produ...
positive regulation of respiratory burst
neutrophil mediated killing of gram-positive bacte...
respiratory burst involved in defense response
positive regulation of interleukin-6 secretion
regulation of granulocyte chemotaxis
positive regulation of myeloid leukocyte cytokine...
response to lipopolysaccharide
cellular response to interleukin-7
response to endoplasmic reticulum stress
response to unfolded protein
defense reponse to fungus
positive regulation of immune response
protein refolding
protein folding
Gene Number
P Value
0.0348
0.0174
0.0000
Rich Factor
M
Camk1d
Prtn3
Elane
Mpo
Expression Level
Ctrl
LIP

Figure S2. Lineage differentiation trajectory of HSPCs and GMP subclusters, related to Figure 2. **(A-C)** Differentiation trajectory generated using Monocle 2. Cells are colored according to cell types (A), pseudotime values (B), and sample (C), respectively. **(D)** Repartition (in percentage) of the different states (1 to 7) of the trajectory for each Seurat cluster. **(E)** Stacked plot of predicted cell types along pseudotime cut into 50 bins for LIP (upper part of the plots) and Ctrl (lower part of the plots) for lymphopoiesis. Black and red stretched lines mark 2 and 1 bifurcation point pseudotime, respectively. **(F-G)** UMAP showing the lineage trajectory of neutropoiesis (F), lymphopoiesis (G). Pseudotime values overlapped on the UMAP. **(H)** TF deviation score of *Cebpa*, *Cebpb*, *Cebpd*, and *Cebpe* in the indicated cell types. **(I-J)** Differentiation trajectory generated using Monocle 2. Cells are colored according to sample (I) and pseudotime values (J). **(K)** UMAP showing the lineage trajectory of GMP subclusters. Pseudotime values overlapped on the UMAP. **(L)** GO:BP enrichment analysis of upregulated genes in the NeuP cluster from LIP versus Ctrl mice. **(M)** Combined violin plots showing marker gene expression in the NeuP of Ctrl and LIP mice.

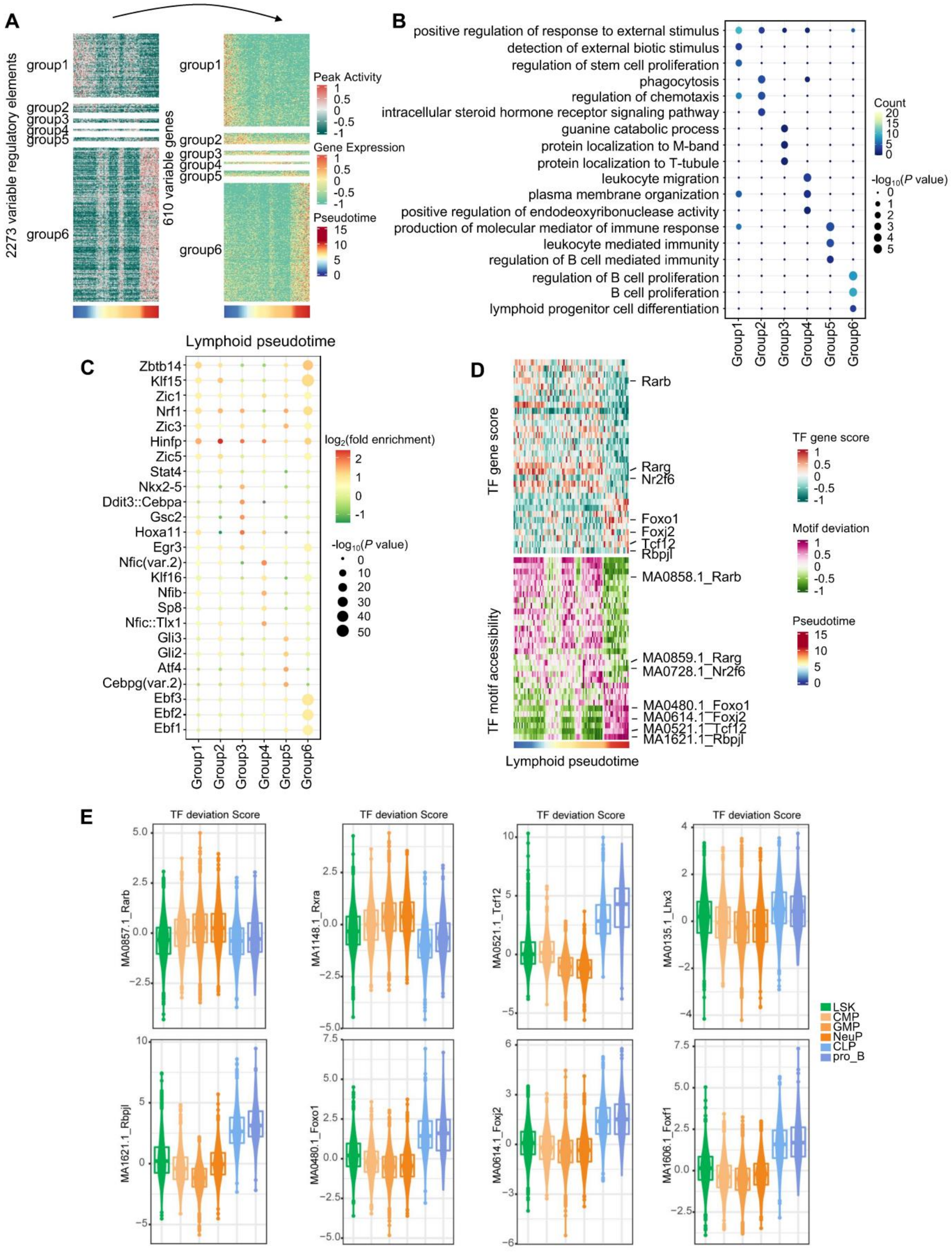

Figure S3. Dynamic molecular features during hematopoietic lineage differentiation, related to Figure 3. (A) Accessibility (left) and expression (right) dynamics across lymphoid

pseudotime. **(B)** GO enrichment terms associated with genes categorized into six interaction clusters. **(C)** Enrichment analysis of TF motifs within peaks corresponding to these six interaction clusters. Color intensity indicates $\log_2$(fold enrichment), while point size reflects $-\log_{10}(P$ value). **(D)** Heatmaps showing the positive TF regulators obtained from the integration of ordered TF gene scores (top) with ordered TF motif accessibility (bottom) across lymphoid pseudotime. **(E)** TF deviation score of *Rarb*, *Rxra*, *Tcf12*, *Lhx3*, *Rbpjl*, *Foxo1*, *Foxj2*, and *Foxf1* in the indicated cell types.

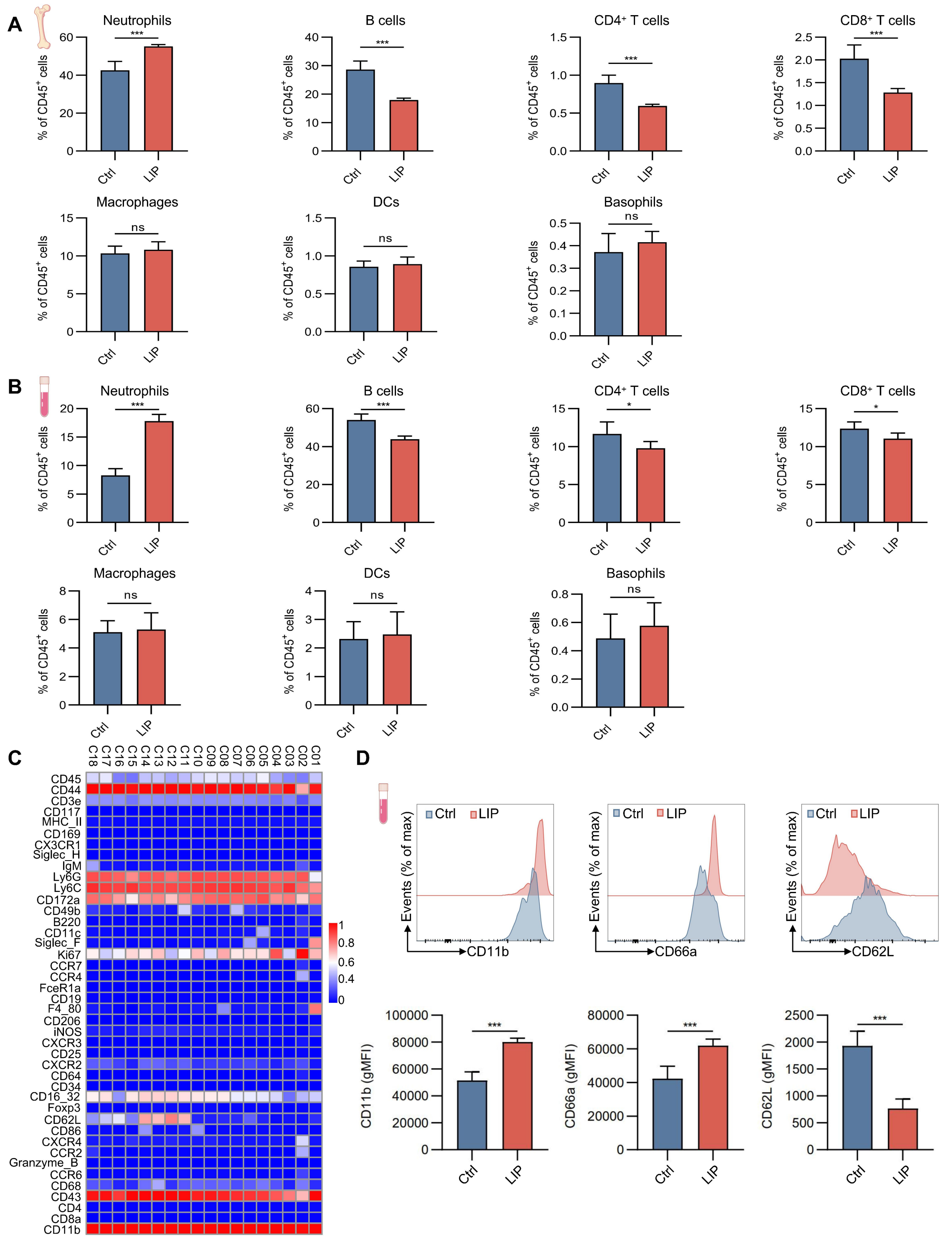

Figure S4. Periodontitis primes neutrophils toward a pro-inflammatory phenotype, related to Figure 4. (A-B) Frequencies of neutrophils, B cells, CD4$^+$ T cells, CD8$^+$ T cells, macrophages, DCs and basophils in CD45$^+$ cells in the BM (A) and PB (B) from Ctrl and LIP

mice. **(C)** Heatmap showing the relative expression level of 42 cell markers within the 18 neutrophil subclusters. **(D)** Flow cytometric analysis of the expression of CD11b, CD66a and CD62L in neutrophils obtained from PB. gMFI, geometric mean fluorescence intensity. Data are presented as the mean ± SD from at least three independent experiments; n = 6 mice/group (A, B, and D). *P* values were calculated using two-tailed Student's *t* test; **P* < 0.05, ***P* < 0.01, ****P* < 0.001, ns indicates no significant difference.

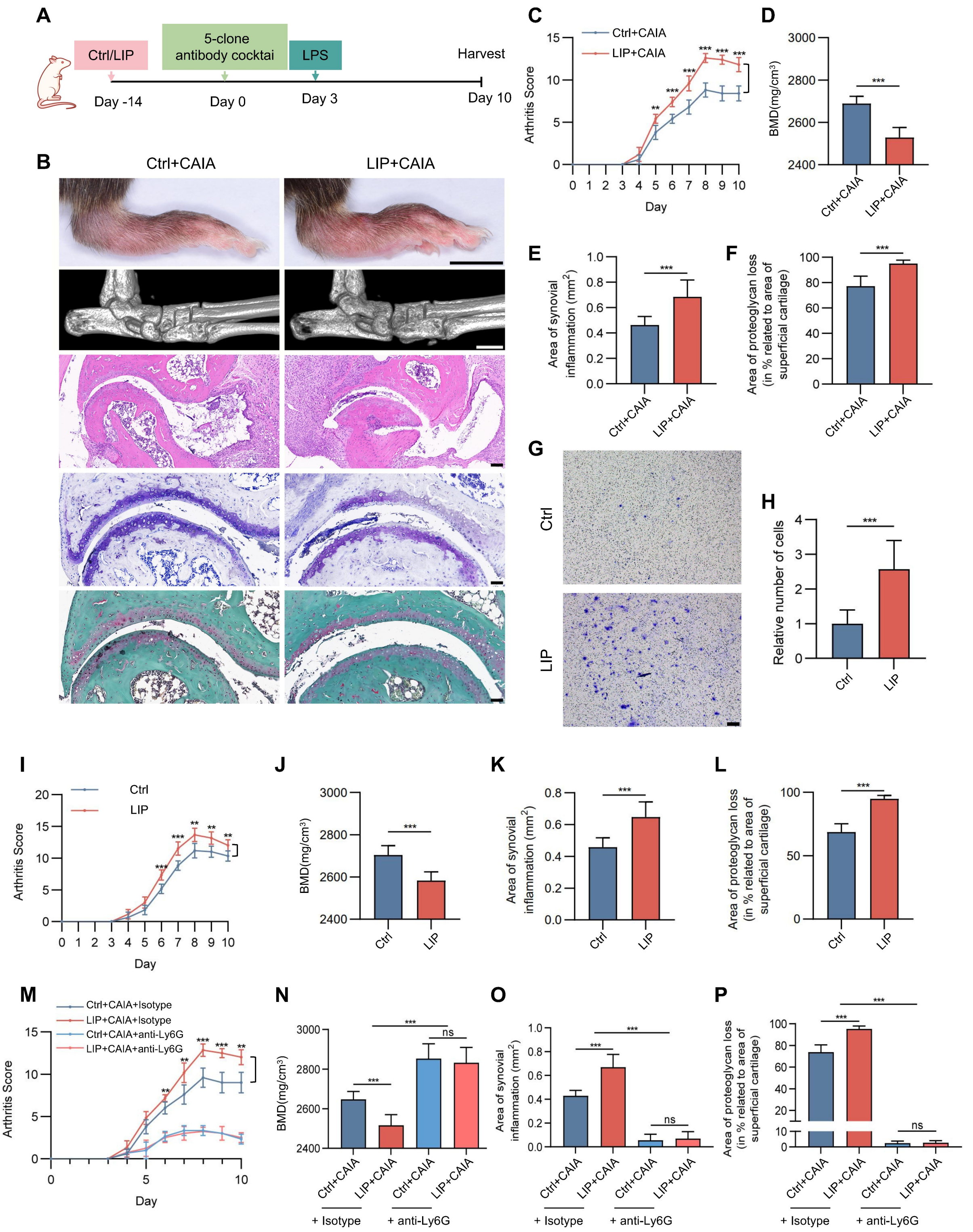

Figure S5. Periodontitis aggravates arthritis by priming neutrophils, related to Figure 5.

(A) Experimental protocol for CAIA model induction in Ctrl or LIP mice. **(B)** Representative

photographs of hindlimbs at the endpoint of the experiment from Ctrl+CAIA and LIP+CAIA groups (scale bar = 5 mm); representative micro-CT images of ankle joints (scale bar = 1 mm); histopathological evaluation of ankle joints was performed using H&E (scale bar = 100 μm), toluidine blue, and safranin-O/fast green staining (scale bar = 50 μm). **(C-F)** Quantitative analysis of arthritis symptoms in Ctrl+CAIA and LIP+CAIA mice, presenting mean arthritis score for the CAIA model (C), BMD (D), synovial inflammation area (E), and proteoglycan loss area (F). **(G)** Representative photomicrographs of neutrophils in the chemotaxis assays (scale bar = 100 μm). **(H)** Quantitative analysis of the chemotaxis assays. **(I-L)** Quantitative analysis of arthritis symptoms in mice treated with neutrophil adoptive transfer for the CAIA model, presenting mean arthritis score for the CAIA model (I), BMD (J), synovial inflammation area (K), and proteoglycan loss area (L). **(M-P)** Quantitative analysis of arthritis symptoms in Ly6G blockade-treated CAIA mice, presenting mean arthritis score for the CAIA model (M), BMD (N), synovial inflammation area (O), and proteoglycan loss area (P). Data are presented as the mean ± SD from at least three independent experiments; n = 6/group (H), n = 8 mice/group (C-F and I-P). P values were calculated using two-tailed Student's *t* test (C-F and H-L) and one-way ANOVA (M-P); $^{*}P < 0.05$, $^{**}P < 0.01$, $^{***}P < 0.001$, ns indicates no significant difference.

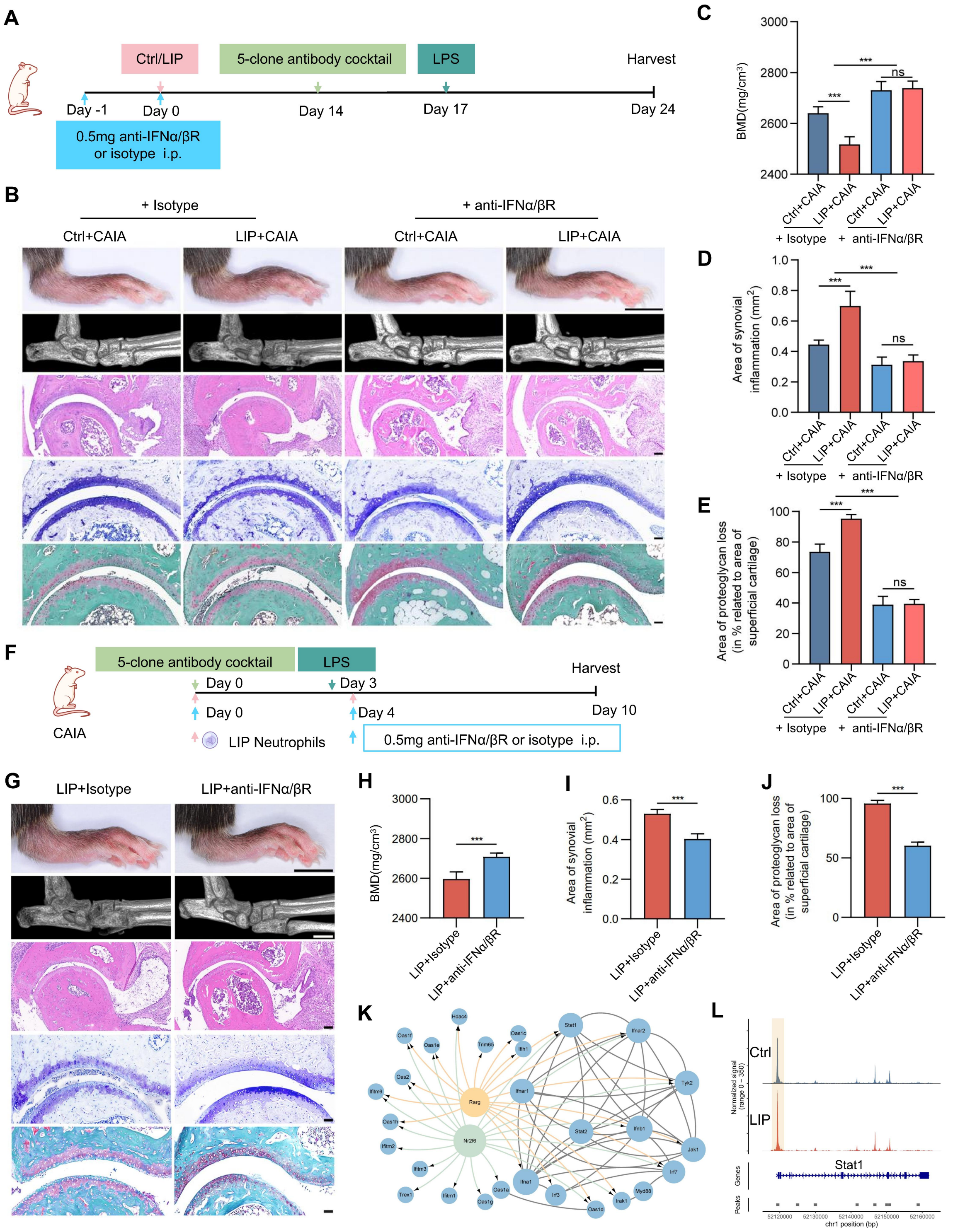

Figure S6. IFN-I signaling blockade alleviates arthritis exacerbated by periodontitis or periodontitis-primed neutrophils, related to Figure 6. (A) Schematic of the experimental design for IFN-I signaling blockade during CAIA model induction. **(B)** Representative photographs of hindlimbs at the endpoint of the experiment from different treatment groups

(scale bar = 5 mm); representative micro-CT images of ankle joints (scale bar = 1 mm); histopathological evaluation of ankle joints was performed using H&E (scale bar = 100 μm), toluidine blue, and safranin-O/fast green staining (scale bar = 50 μm). **(C-E)** Quantitative analysis of arthritis symptoms in CAIA mice treated with IFN-I signaling blockade, presenting BMD (C), synovial inflammation area (D), and proteoglycan loss area (E). **(F)** Schematic of the experimental design for IFN-I signaling blockade during neutrophil transfer experiments. **(G)** Representative photographs of hindlimbs at the endpoint of the experiment from LIP+Isotype and LIP+anti-IFNα/βR groups (scale bar = 5 mm); representative micro-CT images of ankle joints (scale bar = 1 mm); histopathological evaluation of ankle joints was performed using H&E (scale bar = 100 μm), toluidine blue, and safranin-O/fast green staining (scale bar = 50 μm). **(H-J)** Quantification of arthritis symptoms in CAIA mice treated with neutrophil transfer and subsequently subjected to IFN-I signaling blockade, presenting BMD (H), synovial inflammation area (I), and proteoglycan loss area (J). **(K)** Network diagram showing the interaction of the TFs *Rarg* and *Nr2f6* with genes in the type I IFN signaling pathway gene set (GO:0060337). **(L)** Genome browser track showing DARs near promoter regions of *Stat1* locus. Data are presented as the mean ± SD from at least three independent experiments; n = 6 mice/group (C-E and H-J). *P* values were calculated using one-way ANOVA (C-E) and two-tailed Student's *t* test (H-J); $^{*}P < 0.05$, $^{**}P < 0.01$, $^{***}P < 0.001$, ns indicates no significant difference.

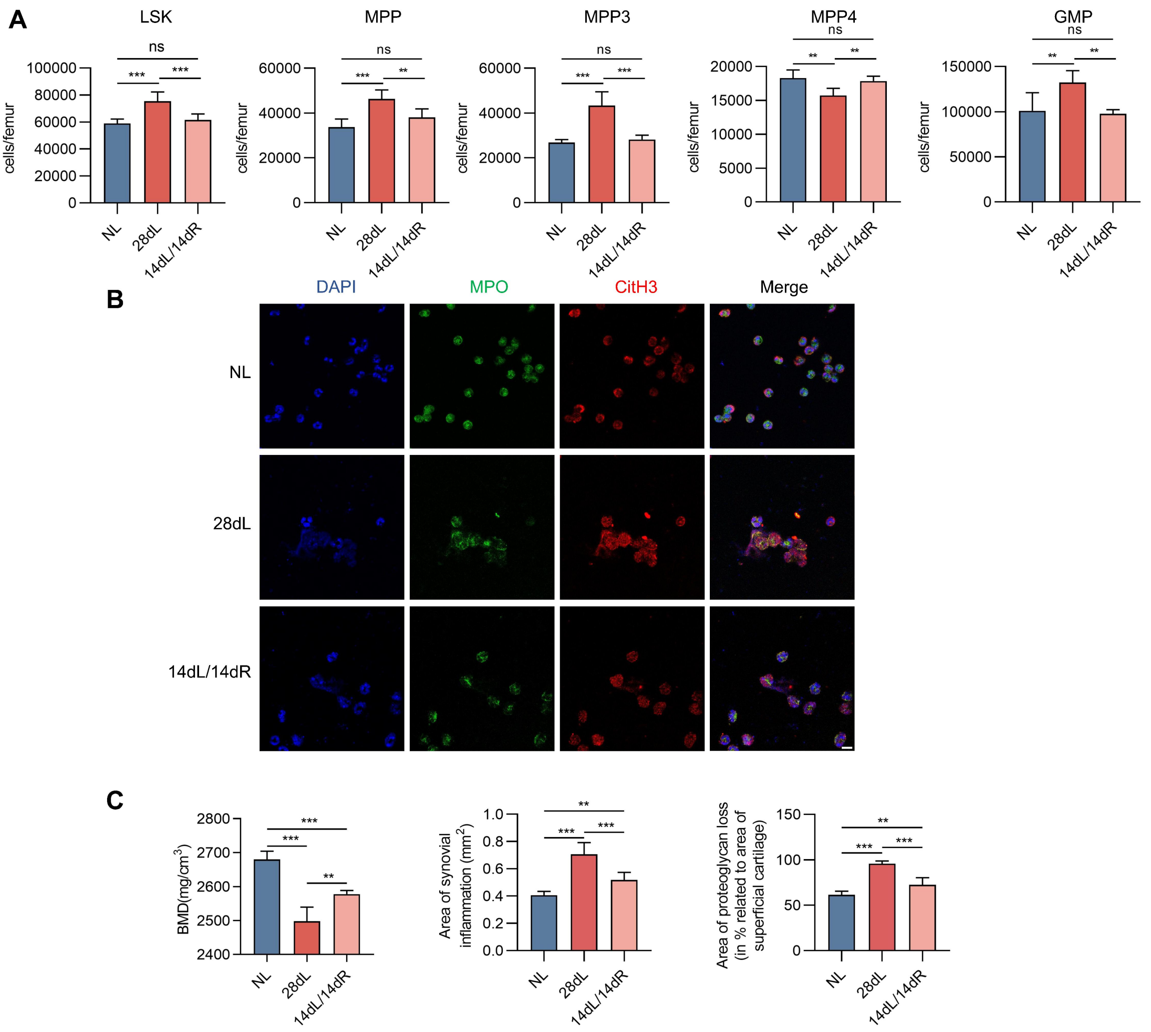

Figure S7. Periodontal inflammation resolution alleviates periodontitis-related arthritis progression, related to Figure 7. (A) Absolute cell numbers of LSK, MPP, MPP3, MPP4 and GMP in total BM cells. **(B)** Representative images of neutrophils stained with citH3 (red), MPO (green), and DAPI (blue). NETs are visualized via colocalization of citH3 and DAPI staining (merged images) (scale bar = 10 μm). **(C)** Quantitative analyses of BMD, synovial inflammation area, and proteoglycan loss area in the ankle joints of NL, 28dL, and 14dL/14dR mice. Data are presented as the mean ± SD from at least three independent experiments; n = 6 mice/group (A and C). *P* values were calculated using one-way ANOVA; *$P < 0.05$, **$P < 0.01$, ***$P < 0.001$, ns indicates no significant difference.

Table S1: TFBStools analysis results for *Nr2f6* and *Rarg* binding sites located within the promoter of *Stat1* gene（EntrezID：20846).

TF	JASPAR_ motifID	Gene	Score	Start	End	Strand	Predicted Sequence
Nr2f6	MA0677.1	*Stat1*	11.9436139334696	387	400	-	CAGATCAAATGTCA
Rarg	MA0860.1	*Stat1*	8.10418915275733	1549	1565	+	GACGTCAGTGGAG GTCC

Table S2. Mouse CyTOF panel.

Metal	Antibody	Clone	Cat number	Company
89Y	CD45	30-F11	103102	Biolegend
113Ln	CD44	IM7	103002	Biolegend
115ln	CD3ε	145-2C11	100302	Biolegend
141Pr	CD117(c-kit)	2B8	105802	Biolegend
142Nd	MHC II(I-A/I-E)	M5/114.15.2	107602	Biolegend
143Nd	CD169(Siglec-1)	3D6.112	142402	Biolegend
144Nd	CX3CR1	SA011F11	149002	Biolegend
145Nd	Siglec H	440c	GTX14268	Genetex
146Nd	IgM	RMM-1	406502	Biolegend
147Sm	Ly-6G	1A8	127602	Biolegend
148Nd	Ly-6C	HK1.4	128002	Biolegend
149Sm	CD172a(SIRPα)	P84	144002	Biolegend
150Nd	CD49b	DX5	108902	Biolegend
151Eu	CD45R(B220)	RA3-6B2	103202	Biolegend
152Sm	CD11c	N418	117302	Biolegend
153Eu	Siglec-F	E50-2440	552125	BD Biosciences
154Sm	Ki-67	SolA15	14-5698-82	ebioscience
155Gd	CD197(CCR7)	4B12	120101	Biolegend
156Gd	CD194(CCR4)	2G12	131202	Biolegend
157Gd	FcεRIα	MAR-1	134302	Biolegend
158Gd	CD19	6D5	115502	Biolegend
159Tb	F4/80	C1:A3-1	MCA497G	Biorad
160Gd	CD206(MMR)	C068C2	141702	Biolegend
161Dy	iNOS	CXNFT	14-5920-82	ebioscience
162Dy	CD183(CXCR3)	CXCR3-173	126502	Biolegend

163Dy	CD25(IL-2Rα)	3C7	101902	Biolegend
164Dy	CD182(CXCR2)	SA044G4	149302	Biolegend
165Ho	CD64(FcγRI)	X54-5/7.1	139302	Biolegend
166Er	CD34	RAM34	553731	BD Biosciences
167Er	CD16/32	93	101302	Biolegend
168Er	Foxp3	FJK-16s	14-5773-82	ebioscience
169Tm	CD62L	MEL-14	104402	Biolegend
170Er	CD86	GL-1	105002	Biolegend
171Yb	CD184(CXCR4)	L276F12	146502	Biolegend
172Yb	CD192(CCR2)	475301	MAB55381-100	R&D Systems
173Yb	Granzyme B	GB11	3173006B	Fluidigm
174Yb	CD196(CCR6)	29-2L17	129802	Biolegend
175Lu	CD68	FA-11	137002	Biolegend
176Yb	CD43	S11	143202	Biolegend
197Au	CD4	RM4-5	100576	Biolegend
198Pt	CD8a	53-6.7	100746	Biolegend
209Bi	CD11b	M1/70	101202	Biolegend

Table S3: The primers used for RT-qPCR.

Gene	Primer sequence (5' to 3') -Forward	Primer sequence (5' to 3')-Reverse
Gapdh	CCATCACCATCTTCCAGG	AGACTCCACGACATACTCA
Ifna1	CTGTGCTTTCCTGATGGTCC	CAGATGAGTCCTTTGATGTGAA
Ifnb1	TGCGTTCCTGCTGTGCT	GTCCGCCCTGTAGGTGAG
Ifnar1	TTCCGAGGTGGGACAATC	CAATAAGAAGGACGAGCAAAGT
